# Dimensional Scaling Effect in Percolative Oxide Semiconductor Transistors

**DOI:** 10.1021/acsnano.5c21838

**Published:** 2026-04-06

**Authors:** Robert Tseng, Yi-Hou Kuo, Yi-Yu Pan, Zheng-Hong Li, Sung-Tsun Wang, Ciao-Fen Chen, Shun-Tsung Lo, Yu-Cheng Chan, Ya-Jing Wu, Shih-Chieh Chen, Cheng-Chen Kuo, Chun-Chen Wang, Cheng-Hsien Wu, Wen-Hsiang Lu, Xinyu Bao, Nguyen Thi Phuong Thao, Emi Minamitan, Ali Javey, Chun-Liang Lin, Der-Hsien Lien

**Affiliations:** † Institute of Electronics, 34914 National Yang Ming Chiao Tung University, Hsinchu 30010, Taiwan; ‡ Electrical Engineering and Computer Sciences, 1438 University of California, Berkeley, California 94720, United States; § Materials Sciences Division, Lawrence Berkeley National Laboratory, Berkeley, California 94720, United States; ∥ Department of Electrophysics, National Yang Ming Chiao Tung University, Hsinchu 30010, Taiwan; ⊥ 63393 Taiwan Semiconductor Manufacturing Company, Ltd., Hsinchu 30010, Taiwan; # Taiwan Semiconductor Manufacturing Company, San Jose, California 95134, United States; ∇ 89266 SANKEN, Osaka University, Ibaraki, Osaka 567-0047, Japan

**Keywords:** percolation transport, amorphous semiconductors, oxide semiconductors, dimensional scaling effect, threshold voltage shift

## Abstract

Percolation transport dominates the charge conduction in amorphous and polycrystalline semiconductors. This study identifies a dimensional scaling effect unique to transistors using percolative semiconductors as channel materials, where the materials’ percolation threshold (*p*_c_) exhibits a strong correlation with the transistor threshold voltage (*V*_T_). We demonstrate that both parameters are fundamentally governed by the semiconductor channel geometry. By reducing channel thickness, width, or length, *p*_c_ is modulated because the availability of conductive pathways is constrained by the channel dimensions, directly driving the observed *V*_T_ shifts. A quantitative link between *p*_c_ and *V*_T_ is established through the percolation potential landscape visualized by scanning tunneling microscopy. The result reveals that the energy landscape is determined by the Fermi level, a characteristic of percolative channels, where device turn-on occurs as the Fermi level exceeds the potential barriers to form conductive pathways. This mechanism is confirmed by temperature-dependent transport measurements, where the extracted activation energies exhibit a strong geometric dependence consistent with the *p*_c_ and *V*_T_ shifts. This scaling effect appears consistently in both n-type In_2_O_3_ and p-type SnO transistors, showing its universality across percolative semiconductors regardless of the carrier type. These findings demonstrate that transport in amorphous semiconductor devices is defined by percolation-governed transport rather than conventional electrostatics or quantum confinement, and establish geometry as a key design parameter for future amorphous electronics.

## Graphical abstract

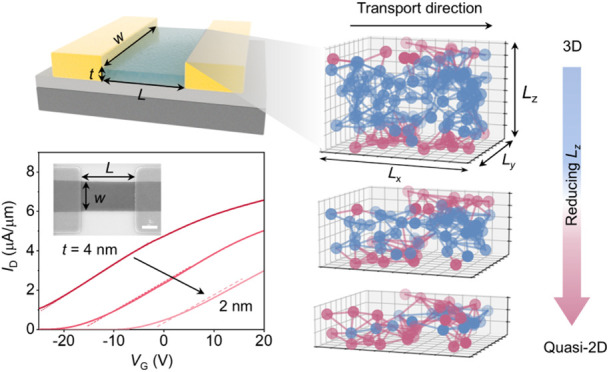

## 1 Introduction

For semiconductor electronics, crystal quality plays a central role in determining device performance, as low defect densities minimize carrier scattering and trap states, enabling high mobility and stable operation. Achieving a high crystalline quality typically requires high-temperature growth to promote crystallinity or postannealing to repair defects. However, these processes often exceed the thermal budgets of emerging integration schemes, particularly heterogeneous and monolithic three-dimensional (3D) integration,^1,2^ where high temperatures can degrade the underlying layers and compromise the integrity of the interconnects. Low-temperature growth, in contrast, generally leads to poor crystalline quality, imposing an inherent trade-off between the processing temperature and device performance. To overcome this limitation, recent efforts have focused on amorphous materials, such as In_2_O_3_, SnO, and a-TeSeO_*x*_,^3–7^ which can sustain high mobility in disordered structures. Their tolerance to structural disorder enables low-temperature deposition by atomic layer deposition and sputtering,^8–10^ allowing conformal growth on complex geometries within strict thermal budgets and making them promising for next-generation integration schemes.^11–13^

On the other hand, structural disorder in amorphous semiconductors leads to device instabilities, notably threshold voltage (*V*_T_) shifts under temperature variations and bias stresses.^14,15^ Moreover, as these transistors are scaled down, *V*_T_ shows a pronounced dependence on the channel thickness, even width, and length. For example, ultrathin amorphous In_2_O_3_ transistors exhibit thickness-dependent *V*_T_ shifts, previously attributed to quantum confinement or electrostatic effects,^16–18^ though the exact origin remains debated. Uncovering such dimension-dependent behavior requires a deeper understanding of the electronic structure and transport mechanisms in the amorphous phase. However, modeling their electronic structure and transport remains challenging due to the absence of periodic order. Unlike crystalline semiconductors, which rely on extended Bloch states and band transport, amorphous materials exhibit transport dominated by disorder through percolation pathways.^19–21^ Meanwhile, their conductivity is governed by the position of the Fermi level (*E*_F_), preserving the tunability characteristics of conventional semiconductors. This gives rise to a semiconductor-percolation duality, in which transport is governed by localized potential barriers imposed by the disordered landscape, while electrostatic modulation of the *E*_F_ controls barrier heights and hence the conductivity.

In this study, we identify a distinct dimensional scaling effect in amorphous semiconductors arising from their percolative transport nature. We show that reducing the device thickness, length, and width modulates the percolation threshold (*p*_c_; defined as the critical fraction of conductive pathways required for long-range connectivity) by constraining the available conductive pathways, which directly drives measurable *V*_T_ shifts. By mapping the potential energy landscape, we establish a quantitative link between *p*_c_ and *V*_T_, revealing that the energy landscape is governed by Fermi level modulation, a defining characteristic of semiconductor-percolation duality. This mechanism is further validated by temperature-dependent transport measurements, where the activation energies exhibit a strong geometric dependence, consistent with the observed *V*_T_ shifts. Consistently observed in both n-type and p-type oxide transistors, our results uncover a geometry-dependent transport behavior that is intrinsic to electronics subjected to percolation mechanisms.

## 2 Results and Discussion

### 2.1 Visualizing Potential Barriers in Amorphous In_2_O_3_ as Percolative Semiconductors

We herein use scanning tunneling spectroscopy (STS) to examine the localized potential of amorphous In_2_O_3_. In_2_O_3_ has emerged as a promising transistor-channel material due to its high mobility in the amorphous phase and compatibility with advanced electronic integration. In_2_O_3_ films were grown by atomic layer deposition on SiO_2_ substrates with electrodes patterned on top of the films. A heavily doped silicon substrate under SiO_2_ serves as the bottom gate to modulate the *E*_F_ of In_2_O_3_ (Figure 1a) and enable the extraction of its transport characteristics. STS measurements were performed using the same device structure by measuring the tunneling current flow from the scanning tip to the contact (device connection for STS measurements in Figure S1). Two representative differential conductance (d*I*/d*V*) profiles obtained from the STS measurements are shown in Figure 1b. Due to the structural disorder in the amorphous semiconductor, multiple midgap states are observed, associated with localized potentials arising from lattice vacancies and defects.^22–24^ Above the gap, high-energy edges are observed, corresponding to mobility edges (*E*_M_) that separate localized midgap states from delocalized states in the conduction band tails. By scanning the semiconductor surface and extracting the energy difference between *E*_M_ and *E*_F_, defined as Δ*E = E*_M_ – *E*_F_, the spatial distribution of the potential barriers can be directly visualized (Figure 1c). As shown in Figure 1d, the observed Δ*E* (∼1.4 eV) arises primarily from local variations in the oxygen content inherent to the amorphous phase, consistent with the density functional theory calculations (Figure S2). The *E*_M_ fluctuation extracted from the spatial mapping is ∼0.5 eV, reflecting the band-edge roughness that is higher than that in crystalline materials due to the structural disorder. Upon ultraviolet exposure,^25^ the *E*_F_ shifts upward, reducing the average Δ*E* from ∼1.4 eV to ∼0.9 eV (Figure 1e), while the intrinsic bandgap remains unchanged, as confirmed by absorption spectroscopy (Figure S3). This decrease in Δ*E*, therefore, reflects a decrease in the effective barrier heights rather than a change in the underlying potential profile. A further increase in *E*_F_ eventually reduces Δ*E* to ∼0.05 eV (Figure 1f), indicating the complete formation of conductive pathways across the percolative semiconductor. This transition corresponds to a marked increase in the electrical conductivity with degenerate semiconductor characteristics (Figure S4). Overall, the observed modulation of barrier height with *E*_F_ reflects the semiconductor-percolation duality of amorphous semiconductors, revealing a key characteristic that distinguishes percolative semiconductors from classical percolative network systems.^26–28^

**Figure 1:**
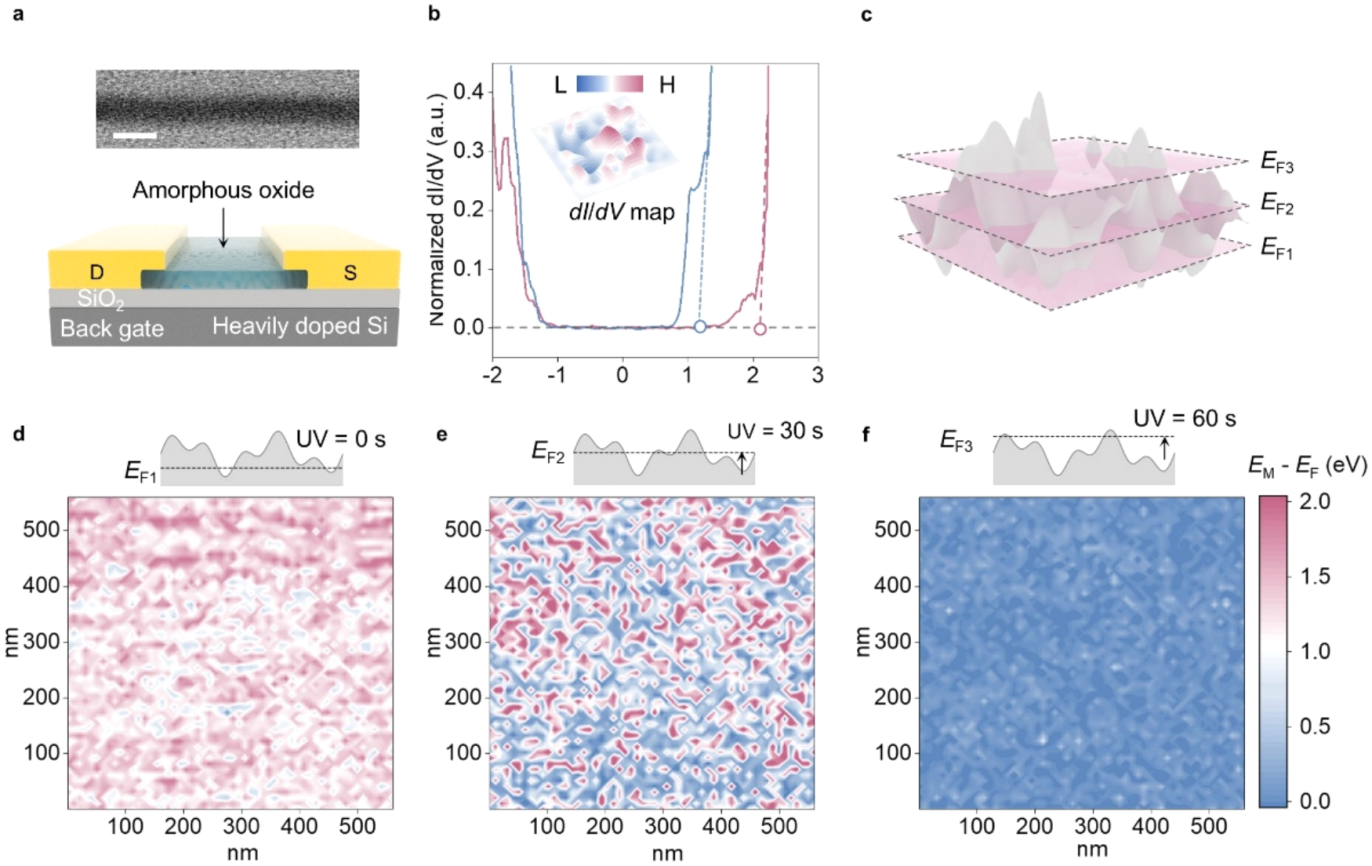
Visualizing percolation barriers in amorphous semiconductors. (a) Back-gated field-effect transistor with an amorphous indium oxide channel on SiO_2_/Si; inset shows a TEM image of the channel (scale bar, 3 nm). (b) d*I*/d*V* spectra of amorphous In_2_O_3_. Two representative curves exhibiting distinctly different spectral features were measured at separate locations on the same sample. The dashed lines indicate *E*_M_. The inset shows the spatial variations in the *E*_M_ across different regions of the material. The full statistical distribution of the energy barriers is provided in Figure S5. (c) Illustration of semiconductor-percolation duality, where transport is barrier-limited, and *E*_F_ remains tunable. (d) Spatial distribution of potential barriers visualized by extracting *E*_M_ – *E*_F_ from the d*I*/d*V* spectra measured at various locations on an as-deposited In_2_O_3_ sample. (e) Reduction in the potential barrier height in In_2_O_3_ upon ultraviolet exposure (30 s), which raises *E*_F_. The emergence of blue regions indicates the formation of low-barrier conductive pathways. (f) Further barrier reduction was achieved by extended ultraviolet exposure (60 s). The inset illustrates the *E*_M_ fluctuations with respect to the varied *E*_F_ levels.

### 2.2 Dimensional Scaling Effect in a Finite-Sized Percolation Transport Model

To investigate the impact of device geometry on charge conduction, we modify classical percolation theory^29^ to construct a transport model incorporating dimensional constraints. Traditional theory assumes an infinite lattice with uniform site connectivity and omnidirectional percolation,^30,31^ where *p*_c_ is primarily determined by the coordination number (*z*) and lattice structures. When percolation models are applied to real devices, finite channel dimensions, including length, width, and thickness, must be considered (Figure 2a). To extend the percolation model to carrier transport in finite lattices, two modifications are needed: (i) boundaries are introduced into the model, where sites near the boundaries exhibit reduced connectivity (reduced *z*) due to lattice termination; and (ii) successful transport is defined as the conductive pathways formed from one side of the lattice to the opposite side, creating a directional percolation current across the medium. These modifications make the lattice dimensions a critical factor in the transport behavior due to the pathway restrictions at the boundaries, as shown in Figure 2b. The amorphous structure is modeled as a quasi-lattice of randomly distributed sites in space, with a total of *L*_*x*_ × *L*_*y*_ × *L*_*z*_ sites (average one site per unit volume), where the simulation parameters *L*_*x*_, *L*_*y*_, and *L*_*z*_ correspond to the physical device length (*l*), width (*w*), and thickness (*t*), respectively, and charge transport is defined along the *x*-axis. Bonds are formed between sites if their separation is below the threshold distance (*d*_th_), which determines the z of each site and the average coordination number (*z̅*) of the whole quasi-lattice (Figure S6). As the effective transport cross-sectional area (*L*_*y*_ × *L*_*z*_) decreases by reducing the thickness *L*_*z*_, *z̅* decreases due to an increased fraction of boundary-adjacent sites with lower connectivity, as shown in Figure 2c. This reduction in *z̅* with decreasing *L*_*z*_ arises from the path confinement effect at the boundaries and is independent of the site connectivity of the model. Even when the overall site connectivity is enhanced by increasing *d*_th_, *z̅* still collapses to surface-limited values as the lattice thickness approaches the two-dimensional limit (Figure 2c).

**Figure 2:**
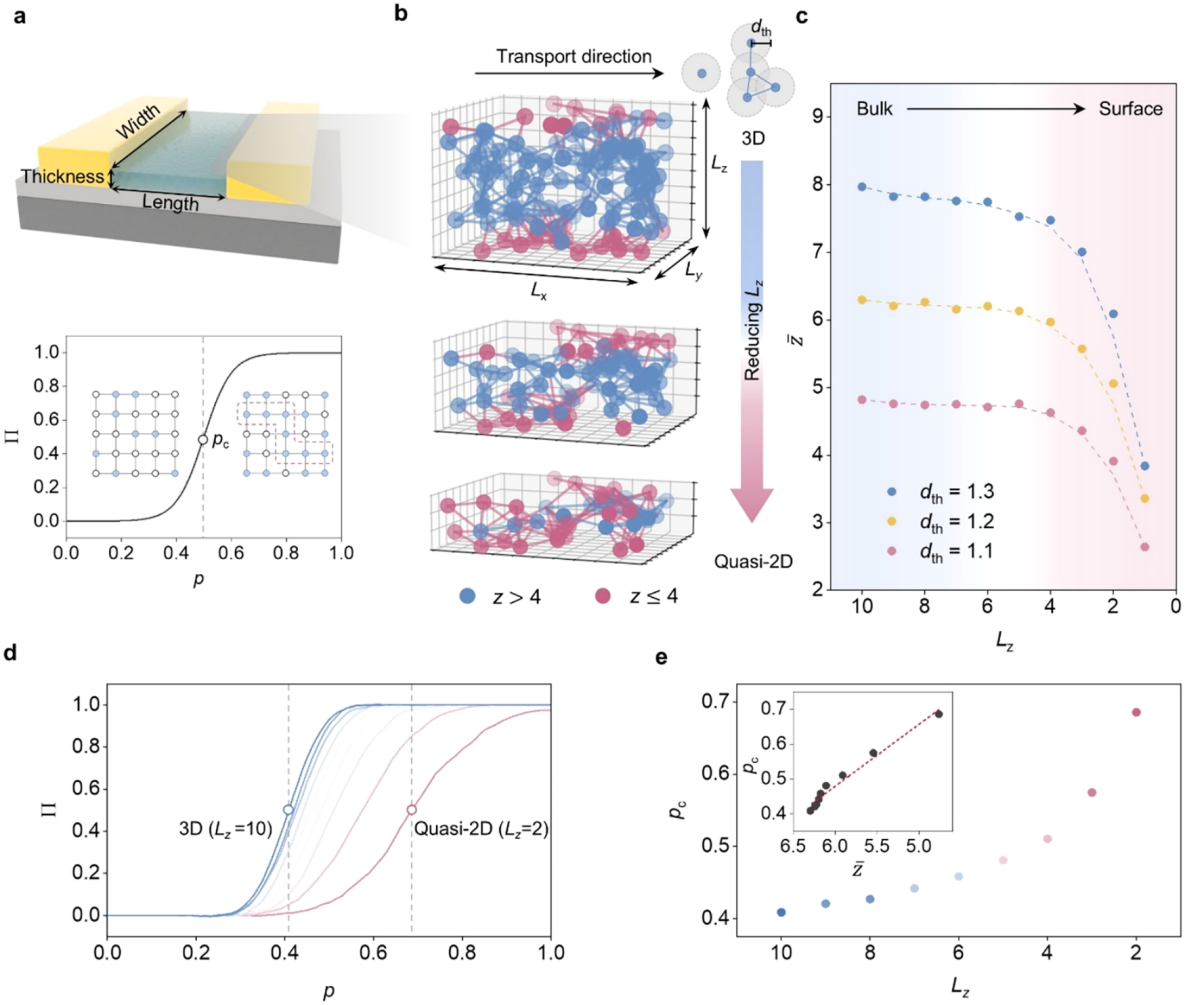
Impact of dimensional scaling on transport paths in a random quasi-lattice. (a) Schematic of a back-gated FET with an amorphous oxide channel, with the length (*l*), width (*w*), and thickness (*t*) indicated. Inset: Definition of the percolation threshold *p*_c_, determined as the occupation probability *p* at which the percolation probability Π reaches 0.5. (b) Schematic of thickness scaling in random lattices representing amorphous semiconductors. Each random site forms a conductive bond with neighboring sites if their separation is less than *d*_th_. Red sites indicate low connectivity (*z* ≤ 4), and blue sites indicate high connectivity (*z* > 4). As the thickness decreases, the increasing ratio of red sites reflects the restricted formation of conductive pathways. (c) Reduction trends of *z̅* with decreasing *L*_*z*_ for various *d*_th_. Simulations are performed on a 10 × 10 × 10 random lattice. As *L*_*z*_ decreases, z̅ decreases from the bulk values to the surface-limited values. (d) Π as a function of *p* for different *L*_*z*_. The Monte Carlo method is employed to determine Π by examining 128 cases for different *p*. Π is defined as the probability of forming a continuous conductive path and is calculated as the ratio of successful percolation cases to the total number of simulated cases. (e) *p*_c_ as a function of *L*_*z*_. The inset shows *p*_c_ with varied *z̅*.

To quantify the path confinement effect, percolation probabilities (Π) as a function of the conducting site fraction (*p*) are calculated using the Monte Carlo method. A typical Π–*p* curve exhibits an abrupt increase in conductivity at the critical threshold of *p*_c_, defined as Π = 0.5, representing the transition from insulating to conductive states (bottom panel of Figure 2a; simulations for different lattice types are summarized in Figure S7–13). While decreasing the lattice thickness from bulk (*L*_*z*_ = 10) toward the nearly two-dimensional limit (*L*_*z*_ = 2), the Π–*p* curves exhibit a rightward shift (Figure 2d), with *p*_c_ increasing from 0.4 to 0.7 (Figure 2e), indicating that a higher fraction of conductive sites is required to achieve percolation. This dimensional dependence demonstrates that the reduced lattice thickness significantly enhances path confinement effects, increasing *p*_c_ owing to a larger fraction of boundary sites with fewer connections, in agreement with the reduced *z̅* (Figure 2e, inset).

### 2.3 Impact of Thickness Scaling on the Threshold Voltage of Percolative Semiconductors

This dimensional dependence revealed by the percolation model is experimentally validated by the analysis of amorphous semiconductor transistors. In_2_O_3_ films with varying thicknesses (*t*) were deposited onto back-gate structures with prefabricated metal contacts to avoid localized annealing from metal deposition,^32^ and the channel length (*l*) and width (*w*) were defined by photolithography. As the *t* of In_2_O_3_ decreases from 4 to 2 nm, the transfer characteristics (*I*_D_–*V*_G_) show a *V*_T_ shift from −34 V to −2 V (Figure 3a,b). In addition to the *V*_T_ shift, the field-effect mobility degrades with decreasing thickness (Figure S14b), which is consistent with percolation arising from dimensional confinement that restricts the connectivity of conductive pathways.

**Figure 3:**
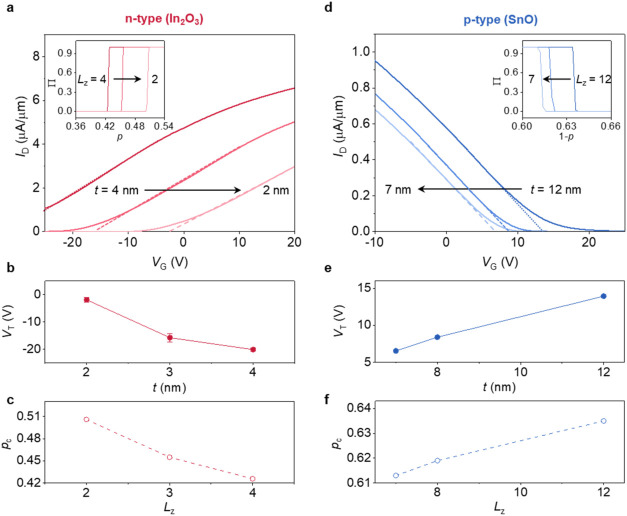
Impact of channel thickness on percolation behaviors and transistor characteristics og n-type and p-type percolative semiconductors. (a) Transfer characteristics of n-type In_2_O_3_ transistors at different channel thicknesses (*t* = 4, 3, 2 nm), with *l* and *w* fixed at 5 μm (see Figure S14a for data on additional thickness). The inset shows the percolation probability (Π) as a function of *p* for the n-type lattice at thicknesses of *L*_*z*_ = 4, 3, 2; *L*_*x*_ = 5000; and *L*_*y*_ = 5000. (b) *V*_T_ with different *t* values for n-type In_2_O_3_. (c) *p*_c_ with different *L*_*z*_ values simulated for the case of n-type semiconductors. (d) Transfer characteristics of p-type SnO transistors at different channel thicknesses (*t* = 12, 8, 7 nm), measured under different *V*_D_ values with *l* and *w* fixed at 5 μm. The inset shows Π as a function of *p* for a p-type lattice at thicknesses of *L*_*z*_ = 12, 8, 7; *L*_*x*_ = 5000; and *L*_*y*_ = 5000. (e) *V*_T_ for p-type SnO with different *t*. (f) *p*_c_ with different *L*_*z*_ simulated for the case of p-type semiconductors.

For an n-type semiconductor, a positive *V*_T_ shift indicates reduced conductivity. A similar trend is observed in the percolation model while matching the model sizes to the aspect ratios of the actual channel size (*l*:*w*:*t* = *L*_*x*_:*L*_*y*_:*L*_*z*_). As *L*_*z*_ of the percolation quasi-lattice decreases from 4 to 2 (with *L*_*x*_ and *L*_*y*_ fixed), *p*_c_ shifts from 0.43 to 0.51 (Figure 3a, inset and Figure 3c), reflecting the enhanced path confinement effect at a reduced thickness. The increase in *p*_c_ with reduced thickness aligns with the observed positive *V*_T_ shift, both indicating reduced electrical conductivity. Previous studies have attributed *V*_T_ shifts in ultrathin In_2_O_3_ transistors to bandgap widening from quantum confinement, which reduces the carrier density and increases *V*_T_ at reduced thickness.^33^ Our findings suggest that the observed *V*_T_ shifts are attributed to the dimensional scaling effect in amorphous semiconductors arising from their percolative transport nature (a behavior consistently observed in sputtered In_2_O_3_; Figure S15).

Here, we establish a quantitative framework linking *p*_c_ and *V*_T_ to bridge the percolation model and experiments. Based on the random band-edge model, the potential barrier landscape is defined as the spatial variation of the *E*_M_,^34^ as observed in the STS mapping. While *E*_F_ is modulated by *V*_G_, the region where the carrier density exceeds a defined threshold is considered conducting and otherwise nonconducting, enabling the determination of *p* at each *V*_G_. By correlating *V*_G_ to *p* and referencing *p*(Π) of quasi-lattices from Monte Carlo simulations, the relationship between *p*_c_ and *V*_T_ can be quantitatively established (Figure S16; details are available in the Supporting Information).

In addition to n-type In_2_O_3_, thickness-dependent *V*_T_ shifts are also observed in ultrathin p-type semiconductors governed by percolative transport. For SnO transistors, a noncrystalline semiconductor also governed by percolation transport, the *I*_D_–*V*_G_ curves exhibit a negative *V*_T_ shift from 14 to 6 V, as *t* decreases from 12 to 7 nm (Figure 3d,e). Although the *V*_T_ shift directions differ between p-type SnO and n-type In_2_O_3_ due to the opposite carrier polarities, both indicate reduced electrical conductivity with decreasing thickness. To simulate p-type transport, *p*_c_ is extracted from Π versus (1–*p*), where successful percolation corresponds to the formation of continuous unbonded pathways, analogous to hole conduction. As a result, *p*_c_ decreases from 0.64 to 0.61, as *L*_*z*_ decreases from 12 to 7 (Figure 3b, inset and Figure 3f), consistent with the *V*_T_ shift. The smaller shift in *V*_T_ and *p*_c_ for p-type SnO stems from both its greater thickness and polycrystalline nature (Figure S17). While a greater thickness reduces confinement effects (Figure 2b), its grain boundary-dominated disorder is also inherently less sensitive to confinement than the atomic-scale disorder in amorphous In_2_O_3_. Despite this difference in magnitude, the consistent trend in both systems validates the universality of the percolation mechanism in disordered semiconductors.

### 2.4 Impact of Length and Width Scaling on the Percolation Behavior

In addition to thickness, scaling the device *l* and *w*, which correspond to *L*_*x*_ and *L*_*y*_ in the percolation model, also affects percolation transport (Figure 4a). Specifically, when *L*_*y*_ and *L*_*z*_ are fixed, shortening the transport length *L*_*x*_ increases the probability of forming a continuous path, thereby lowering *p*_c_ (Figure 4b). Conversely, since both *L*_*y*_ and *L*_*z*_ are perpendicular to the transport direction, reducing either of them constrains the available pathways. At fixed *L*_*x*_ and *L*_*z*_, reducing *L*_*y*_ narrows the channel available for percolation, thus also increasing *p*_c_ (Figure 4c). Notably, significant shifts in *p*_c_ occur only when *L*_*x*_ and *L*_*y*_ decrease to a critical threshold (∼10^3^), at which path confinement becomes prominent.

**Figure 4:**
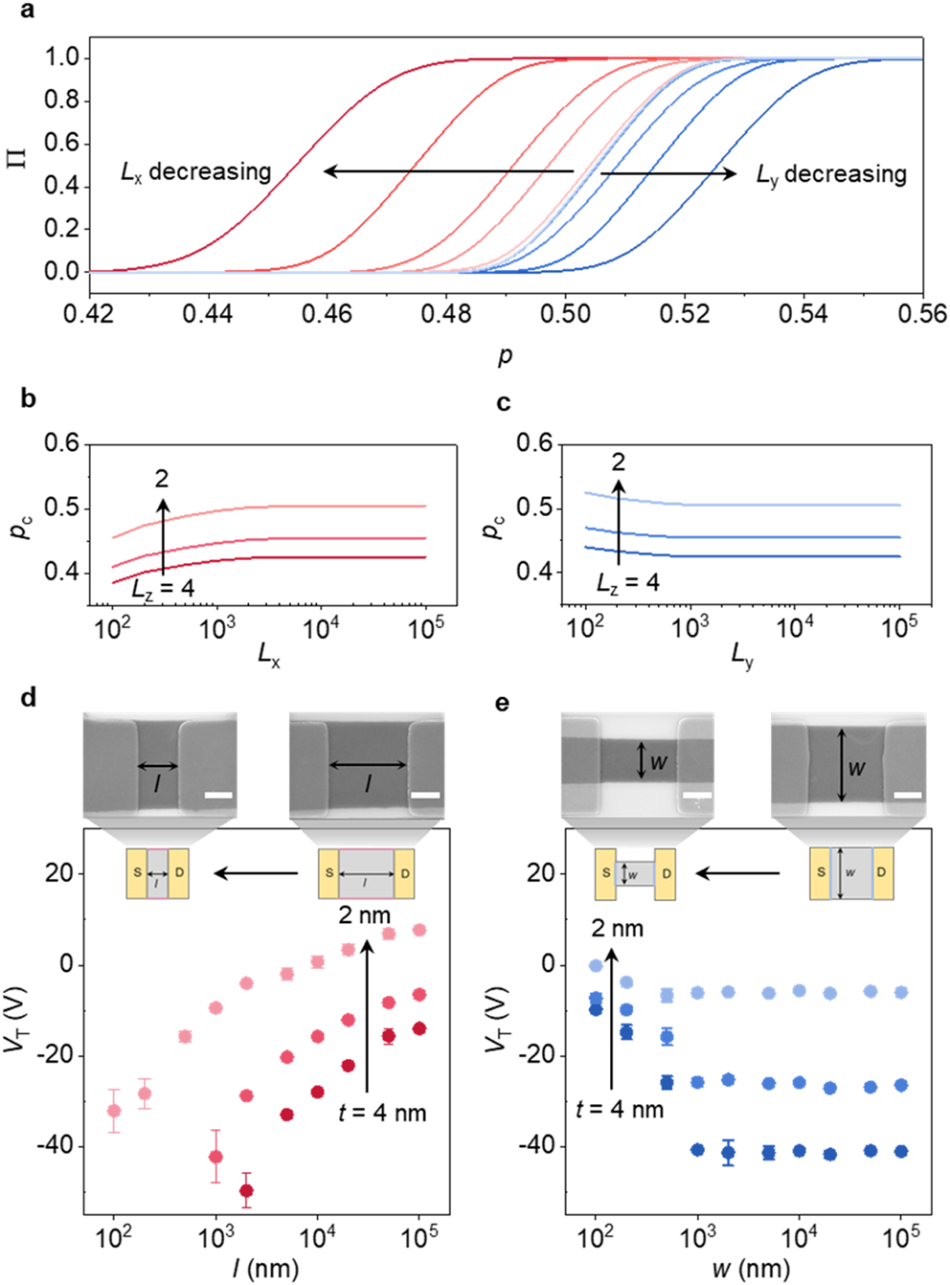
Effect of channel length and width scaling on percolation behavior and threshold voltage. (a) Percolation probability Π as a function of occupation probability *p* for lattices with *L*_*z*_ = 3. The percolation threshold *p*_c_ shifts with decreasing lattice length *L*_*x*_ (red) and width *L*_*y*_ (blue). (b) Simulated *p*_c_ as a function of *L*_*x*_ for different *L*_*z*_ values at fixed *L*_*y*_ = 5000. (c) Simulated *p*_c_ as a function of *L*_*y*_ for different *L*_*z*_ at fixed *L*_*x*_ = 5000. (d) Threshold voltage *V*_T_ of In_2_O_3_ transistors with varying channel lengths *l* at a fixed width *w* = 5 μm. Insets: SEM images of short- and long-channel devices (scale bar, 3 μm). (e) Threshold voltage *V*_T_ of In_2_O_3_ transistors with varying channel widths *w* at a fixed length *l* = 5 μm. Insets: SEM images of narrow- and wide-channel devices (scale bar, 3 μm).

The shifts in *p*_c_ caused by the scaling of *L*_*x*_ and *L*_*y*_ are similarly reflected in the *V*_T_ shift of the transistors. To investigate this scaling effect, *V*_T_ is extracted for In_2_O_3_ transistors with a fixed *t* of 2 nm and varying *l* and *w*. Reducing *l* results in a negative *V*_T_ shift, in agreement with the decreased *p*_c_ when *L*_*x*_ is reduced, indicating the enhanced ease of forming continuous conduction pathways (Figure 4d). In contrast, decreasing *w* leads to a positive *V*_T_ shift, reflecting the increased difficulty in forming continuous pathways (Figure 4e). These opposing trends are unlikely to originate from interface effects, as the interface trap density should be uniform for a fixed thickness (transmission electron microscopy and energy dispersive X-ray spectroscopy analyses in Figure S18 rule out artifacts from fabrication-induced edge defects). Furthermore, we observe no clear correlation between the subthreshold swing (SS) and *V*_T_ shifts (Figure S19), indicating that the shifts are an intrinsic property of the percolation network. Note that *V*_T_ shifts from the *l* and *w* scaling are less pronounced than those from the *t* scaling, as the thickness remains the most confined dimension and is the primary factor governing percolation transport. In contrast to the conventional short-channel and narrow-width effects arising from electrostatic coupling at nanoscale dimensions,^35,36^ *V*_T_ shifts in amorphous percolative semiconductors originate intrinsically from the path confinement effect.

### 2.5 Temperature-Dependent Transfer Characteristics of the Percolative Channels

Temperature-dependent transport measurements were performed for devices with channel thicknesses of 2–4 nm, as shown in Figure 5a–c. As the temperature decreases, the drain current drops across all devices due to the thermally activated nature of the carrier transport across the potential barriers. To elucidate the transport dynamics, Arrhenius plots (ln­(*I*_D_) vs T^–1^) were extracted for various *V*_G_, as shown in the insets of Figure 5a–c. The linear relationship observed in the Arrhenius analyses indicates that charge transport is governed by a barrier-limited landscape inherent to the disordered amorphous material rather than simple band-like conduction. The activation energies (*E*_a_) in Figure 5d, extracted from the slopes of the Arrhenius plots, reveal distinct transport regimes across different *V*_G_ ranges. At low *V*_G_ (subcritical region), the extraction of *E*_a_ is hindered by the absence of a continuous conductive path. Near *V*_T_ (critical percolation region), *E*_a_ reaches a maximum (*E*_a,max_), corresponding to the “bottleneck” barrier of the initial percolation path. As *V*_G_ further increases (supercritical region), the device is turned on as the *E*_F_ suppresses the local barriers and creates multiple parallel pathways, reducing the effective *E*_a_. Such nonmonotonic behavior provides a unique signature of percolation transport.

**Figure 5:**
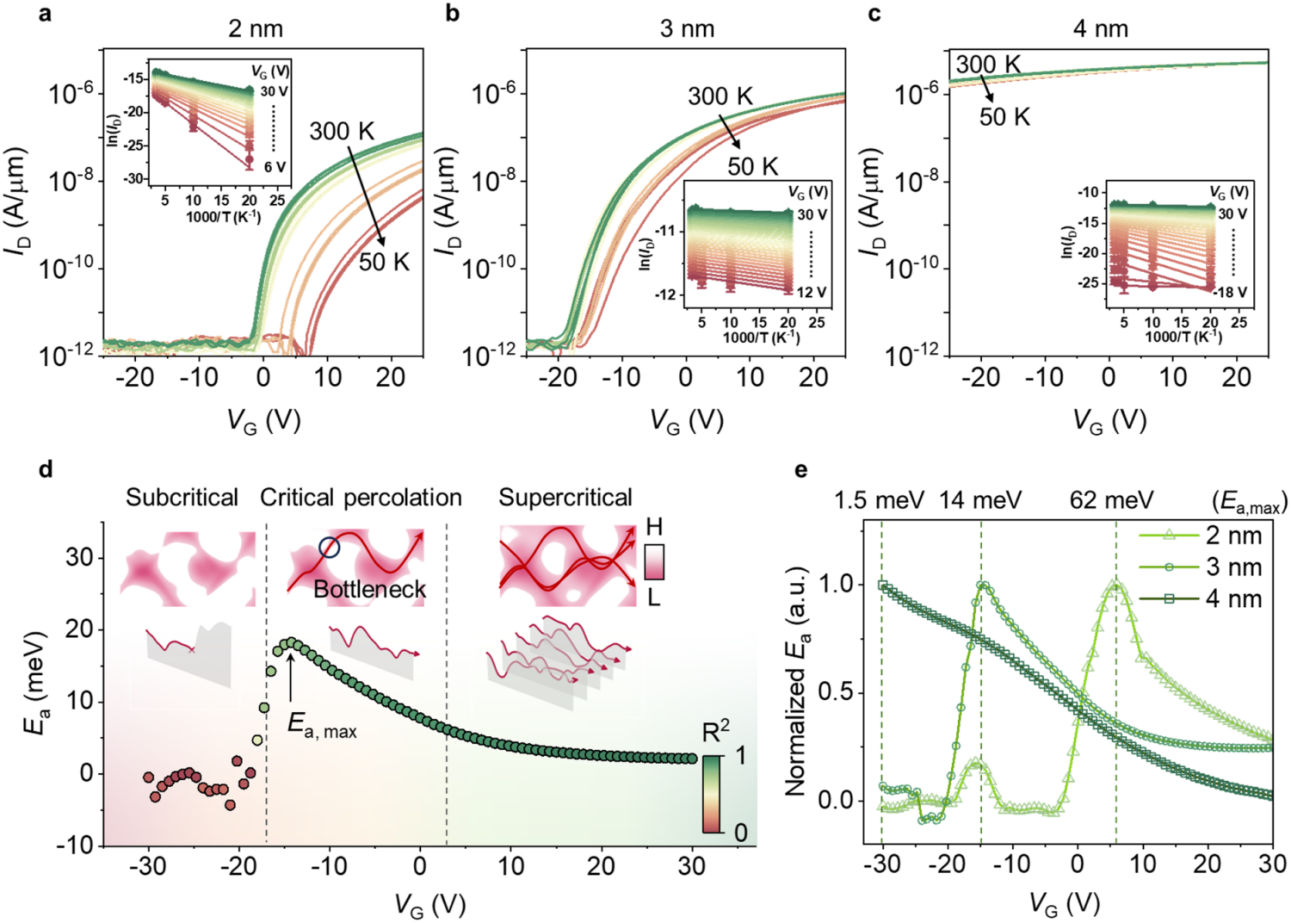
Temperature-dependent transport in devices with different thicknesses. (a–c) Temperature-dependent transfer characteristics of amorphous In_2_O_3_ transistors with channel thicknesses of 4, 3, and 2 nm, respectively, measured from 300 K to 50 K. The insets show the corresponding Arrhenius plots at various gate voltages. (d) *V*_G_-dependent activation energy (*E*_a_) extracted for 3 nm devices. The peak *E*_a_ increases in magnitude and shifts to a higher *V*_G_ as the channel thickness is reduced. Insets schematically illustrate the transport regimes: isolated conductive regions (left), formation of a critical percolation path through a bottleneck (middle), and a well-percolated network (right). *R* ^2^ represents the coefficient of determination, which quantifies the linearity of the Arrhenius relationship. (e) *V*_G_-dependent normalized activation energy (*E*_a_/*E*_a,max_) for devices with 4, 3, and 2 nm thicknesses. The vertical dashed lines mark the gate voltage position of *E*_a,max_ for each curve, with the corresponding absolute energy values annotated above.

The dependence of *E*_a_ on the channel thickness is shown in Figure 5e. As the thickness decreases, the *E*_a_–*V*_G_ profiles shift, and *E*_a,max_ moves toward more positive *V*_G_ values, consistent with the *V*_T_ shifts observed for the corresponding thicknesses. The *E*_a,max_ values also increase from 1.5 to 62 meV as the thickness is reduced from 4 to 2 nm, reflecting an enhanced bottleneck barrier while the pathways are limited with reduced thicknesses. Similarly, varying *l* and *w* shifts the *E*_a_–*V*_G_ profiles, consistent with the observed *V*_T_ trends (Figure S20). Reducing *l* shifts the *E*_a_–V_G_ profiles negatively, indicating easier pathway formation, while decreasing *w* shifts it positively, reflecting increased transport difficulty. Similarly, these scaling effects are less pronounced than those from thickness scaling because thickness is the most confined dimension (down to 2 nm), whereas the length and width remain in the micrometer to hundred-nanometer range. This systematic modulation of *E*_a_ across all three dimensions provides definitive evidence that transport is governed by geometric path confinement.

## 3 Conclusion

Quantum confinement effects are commonly observed in low-dimensional materials such as quantum wells and two-dimensional materials, leading to bandgap widening and altering charge transport.^33,37–39^ In amorphous semiconductors, however, such bandgap widening is not evident as the thickness approaches the two-dimensional limit, likely due to broad band-tail states (Figure S21). Instead, we show that carrier transport in dimensionally scaled amorphous devices is primarily governed by percolation, where *V*_T_ shifts originate from path confinement effects, a mechanism distinct from that in conventional semiconductors. This claim is supported by temperature-dependent transport measurements, which reveal an increase in *E*_a_ as the channel thickness decreases. The observed geometry-driven effect is not limited to thickness but also appears with changes in the device length and width. Unlike electrostatic short-channel effects that emerge only at nanoscales, percolation-induced *V*_T_ shifts occur even at micrometer scales. This behavior is consistently observed in both n-type and p-type percolative semiconductors as well as in films prepared by different deposition methods, confirming its generality. These findings emphasize the importance of incorporating percolation physics into the amorphous device design, providing a strategy to modulate the threshold voltage by synergistically optimizing the geometry or employing controlled doping, and opening new possibilities for geometry-driven modulation beyond conventional scaling.

## 4 Experimental Section

### 4.1 In_2_O_3_ Device Fabrication

Devices were fabricated on heavily doped Si substrates with a 50 nm thermally grown SiO_2_ layer serving as a global back-gate. Electron-beam lithography was first used to define the source/drain regions, followed by electron-beam evaporation of 40 nm Ni and a lift-off process to form the ohmic contacts. In_2_O_3_ thin films of varying thicknesses were then deposited by atomic layer deposition (ALD) at 250 °C using trimethylindium (TMIn) and O_3_ as indium and oxygen precursors, respectively. The active channel regions were patterned via electron-beam lithography and defined using Ar plasma dry etching to achieve precise control over the channel width and length. Finally, the device’s active area was patterned to ensure accurate electrical characterization.

### 4.2 SnO Device Fabrication

Device fabrication began with a standard wet and dry precleaning of 300 mm Si substrates. A 50 nm SiO_2_ dielectric layer was grown using a furnace thermal oxidation process. SnO thin films were then deposited by sputtering, providing an active semiconductor layer. The width/length (*w*/*l*) combinations of the channels were first defined by e-beam lithography, followed by the deposition and patterning of source/drain metal contacts using 30 nm of Au and 30 nm of Ni via electron-beam evaporation and lift-off, respectively. The active areas of the SnO films were defined by dry etching with SF_6_ gas. Finally, the fabricated devices underwent postannealing in an N_2_/O_2_ ambiance at 250 °C for 1 h to enhance the device performance.

### 4.3 Device Characterization

The thickness of the as-grown In_2_O_3_/SnO films was measured using transmission electron microscopy (TEM) analysis. Cross-sectional specimens were prepared using a focused ion beam (FIB) system (Auriga, Carl Zeiss) and examined with a TEM (none-Cs Metrios). Electrical measurements were performed using an Agilent B2902B source. The field-effect mobility (μ_FE_) and threshold voltage (*V*_T_) of the ultrathin In_2_O_3_ transistors were extracted in the linear regime using the conventional MOSFET equation for *V*_D_ ≪ *V*_G_ – *V*_*T*_: $I_{D}=\frac{W}{L}\mu_{\mathrm{FE}}C_{\mathrm{ox}}\left(V_{G}-V_{T}\right)V_{D}$ , where *C*_ox_ is the oxide capacitance. The resulting field-effect mobility (μ_FE_) was obtained from $\mu_{\mathrm{FE}}=\frac{Lg_{m}}{WC_{\mathrm{ox}}V_{D}}$ , where *g*_m_ is the maximum transconductance. The resulting threshold voltage (*V*_T_) was determined by linear extrapolation from *I*_D_ versus *V*_G_, using the maximum *g*_m_ point and extrapolating to *I*_D_ = 0, with *V*_D_/2 added to obtain the intercept on the *V*_G_-axis.

### 4.4 Monte Carlo Simulations

Monte Carlo percolation simulations were performed to model charge transport in amorphous semiconductors under dimensional scaling. The simulation grid was designed to mirror the aspect ratio of the device channel, with the lattice dimensions proportionally scaled to match the experimental transistor’s channel length (*l*), width (*w*), and thickness (*t*). A scale of 1 nm to 1 lattice unit was employed to maintain the geometric aspect ratio of the device within the simulation while also aligning with the scale of potential fluctuations observed in our STS measurements. As a result, different simulation runs employed distinct grid sizes, ensuring that the percolation characteristics remained representative of the corresponding physical system. Each simulation trial involved a randomly generated site percolation network, where sites were assigned as either conductive or insulating based on the conducting site fraction (*p*). To ensure statistical reliability, 128 independent trials were performed for each set of conditions, allowing an accurate estimation of the percolation threshold (*p*_c_), defined as the value of *p* at which the percolation probability (Π) reaches 0.5. To determine whether a percolation path exists across the system, we implemented a Breadth-First Search (BFS) algorithm.^40^ The BFS algorithm systematically explores the connectivity of conducting sites, starting from one side of the grid and attempting to reach the opposite boundary. If a continuous path of conducting sites is found, then the system is considered to be percolated. Boundary conditions were applied such that sites beyond the defined grid size were considered edge boundaries and excluded from participating in the conduction.

## Supplementary Material



## Data Availability

All image data and processed numerical values supporting the findings of this study are available at Zenodo 10.5281/zenodo.18706319. The custom code used for percolation lattice simulations and data analysis is available at Zenodo 10.5281/zenodo.16741296. Additional data sets are available from the corresponding author upon reasonable request.

## Notes

Detailed descriptions of *ab initio* molecular dynamics and density-of-states calculations for amorphous In_2_O_3_; percolation simulations addressing dimensionality, coordination number, and finite-size effects; construction of random lattice models; derivation of the percolation-based transport framework, linking the conducting site fraction to threshold voltage; additional optical characterization, device transfer characteristics; schematic of the STM measurement setup; transfer characteristics of the In_2_O_3_ device before and after UV exposure; X-ray diffraction (XRD) patterns; Tauc plot for In2O3 films of varying thicknesses (Figures S1–S21) (PDF)

## Abbreviations

2D: two-dimensional
3D: three-dimensional
*V*_T_: threshold voltage
*V*_G_: gate voltage
*p*_c_: percolation threshold
*E*_M_: mobility edge
*E*_F_: Fermi level
