# Supplementary material for: Dimensional Scaling Effect in Percolative Oxide Semiconductor Transistors
Source: ACS Nano. 2026 Apr 6;20(15):11756–64. doi: 10.1021/acsnano.5c21838 (PMC13104172; doi:10.1021/acsnano.5c21838)
Supplement: Supplementary file 1 [file nn5c21838_si_001.pdf]

Supporting Information

Dimensional scaling effect in percolative oxide semiconductor transistors

Robert Tseng[1, 2, 3], Yi-Hou Kuo[4], Yi-Yu Pan[1], Zheng-Hong Li[4], Sung-Tsun Wang[1], Ciao-Fen Chen[4], Shun-Tsung Lo[4], Yu-Cheng Chan[1], Ya-Jing Wu[1], Shih-Chieh Chen[1], Cheng-Chen Kuo[5], Chun-Chen Wang[5], Cheng-Hsien Wu[5], Wen-Hsiang Lu[5], Xinyu Bao[6], Nguyen Thi Phuong Thao[7], Emi Minamitan[7], Ali Javey[2, 3]*, Chun-Liang Lin[4]*, Der-Hsien Lien[1]*

[1]Institute of Electronics, National Yang Ming Chiao Tung University, Hsinchu 30010, Taiwan

[2]Electrical Engineering and Computer Sciences, University of California, Berkeley, California 94720, United States

[3]Materials Sciences Division, Lawrence Berkeley National Laboratory, Berkeley, California 94720, United States

[4]Department of Electrophysics, National Yang Ming Chiao Tung University, Hsinchu 30010, Taiwan

[5]Taiwan Semiconductor Manufacturing Company, Ltd., Hsinchu 30010, Taiwan

[6] Taiwan Semiconductor Manufacturing Company, San Jose, California 95134, United States

[7]SANKEN, Osaka University, Ibaraki, Osaka 567-0047, Japan

*Address correspondence to: dhlien@nycu.edu.tw; clin@nycu.edu.tw; ajavey@berkeley.edu

The file includes:

Supplementary Text

Supplementary Fig. 1-21

References 1-13

Supplementary Text

<u>DOS Computation</u>

The amorphous structures were generated using melt-quench simulations with *ab initio molecular dynamics* (AIMD),[1] implemented using the *Vienna Ab initio Simulation Package* (VASP).[2, 3] The generalized gradient approximation (GGA) with the Perdew–Burke–Ernzerhof (PBE) function[4, 5] was also applied. For AIMD simulations, the energy cutoff was set to 260 eV. In the static density of states (DOS) calculations, the hybrid-functional method implemented in VASP was used, with a cutoff energy of 400 eV.[6, 7] Before the DOS calculation, atomic positions were relaxed until the Hellmann–Feynman forces on each atom were below 0.01 eV/Å.

The bixbyite In_2O_3 unit cell, containing 80 atoms, was used as the initial structure. Oxygen vacancies were introduced into the system to explore various stoichiometries. Quench rates reported in prior studies[8] were adopted to generate amorphous In_2O_3 (a-In_2O_3). The melt-quench process involved melting the system at 3000 K, followed by gradual cooling to the melting point (2000 K) at a rate of 100 K/ps. The system was then further cooled to 100 K at a rate of 200 K/ps. Finally, the amorphous structure was equilibrated at 300 K for 6 ps. A k-point grid of 2×2×2 was used throughout the AIMD simulations.

For the DOS calculations, the k-point grid was increased to 4×4×4, and a Gaussian broadening factor of 0.01 eV was applied. The obtained DOS results are shown in Figure S2.

In systems with oxygen vacancies, shifts in the Fermi level were observed as previously reported.[9] These shifts can be attributed to excess carriers introduced by the vacancies.[10] It should be noted that the relatively small system size used in these simulations may limit the accuracy of the absolute Fermi level position; however, the predicted trends can be compared with experimental results.

Finite system size effects in percolation models

The influence of coordination number on percolation threshold was systematically analyzed by transitioning from two-dimensional (2D) to three-dimensional (3D) percolation models. In percolation theory, the coordination number, defined as the number of neighboring sites that each site can connect to, plays a crucial role in determining the percolation threshold (p_c). In lower-dimensional systems, such as purely 2D networks, each site has a limited number of connections (e.g., four in a square lattice with nearest-neighbor connectivity), which raises the critical fraction of conducting sites required for a continuous percolation path to form. As the system transitions to quasi-2D and then to fully 3D structures, additional transport pathways emerge, effectively lowering p_c by increasing the probability of forming a continuous conduction network. The Monte Carlo simulations conducted in this study illustrate this trend by varying the lattice dimensions along different axes and examining the resulting percolation probability distributions.

The influence of coordination number (z) on the p_c is presented (Figure S7-10). A 2D nearest-neighbor percolation system exhibits a high p_c due to limited connectivity (Figure S7). The addition of diagonal connections enhances z and decreases p_c (Figure S8). This analysis is further extended to 3D systems, where additional transport pathways further reduce p_c (Figure S9 and 10). The relationship between z and p_c in regular lattices demonstrates that increasing z lowers p_c due to enhanced connectivity (Figure S11). However, in random lattices, structural disorder influences the effective z, which in turn affects p_c. The percolation simulations were performed in a random lattice with different system sizes (Figure S12). A comparison between regular and random lattices shows that, unlike regular lattices where the transition from a non-percolating to a percolating state is sharp, random lattices exhibit a more gradual transition due to structural disorder, which affects connectivity and delays the formation of a spanning cluster (Figure S13).

Correlation between p_c and V_T

To determine the conducting-site fraction (p) as a function of gate voltage (V_G), we model the disordered semiconductor channel using a three-dimensional random band-edge[11] (mobility-edge) landscape. Each spatial site (i, j, k) is assigned a local mobility-edge energy E_M (i, j, k), drawn from a Gaussian distribution,

$$g(E_M) = \frac{1}{\delta_0\sqrt{2\pi}} \exp\left(-\frac{(E_M-\mu)^2}{2\delta_0^2}\right)$$

where δ_0 characterizes the amplitude of energetic disorder and μ represents the average position of the mobility edge. The parameter μaccounts for the mean band-edge offset of the disordered system, while δ_0 captures spatial fluctuations around this mean value. Together, these parameters

define a three-dimensional potential landscape with both global energy alignment and local disorder.

For a given realization of E_{M} (*i, j, k*), the local three-dimensional carrier density at each site is calculated as a function of the Fermi level (E_{F}),

$$n(i,j,k;E_F) = \int_{-\infty}^{\infty} D(E,E_M)\, f(E,E_F)\, dE,$$

where $f(E, E_F)$ is the Fermi–Dirac distribution. The density of states $D(E, E_{\mathrm{M}})$ consists of extended states above the mobility edge and localized tail states below it,

$$D(E,E_M) = \begin{cases} D_{C0}\sqrt{(E-E_M)+(N_m/D_{C0})^2}, & E > E_M, \\ N_m \exp\left(\frac{E-E_M}{E_0}\right), & E < E_M. \end{cases}$$

Here, D_{C0} is the conduction-band DOS prefactor, N_{m} is the density of localized tail states, and E_0 is the characteristic energy width of the exponential tail states.

Because E_{M} (*i, j, k*) varies spatially in three dimensions, the resulting carrier-density distribution is highly inhomogeneous, giving rise to a complex network of conducting and non-conducting regions throughout the volume. A site is defined as conducting when its local carrier density exceeds a critical threshold n_{th}. The conducting-site fraction (p) is therefore given by

$$p(E_F) = \frac{1}{N}\sum_{i,j,k} \Theta\,[n(i,j,k;E_F) - n_{\mathrm{th}}]$$

Where Θ is the unit step function and N is the total number of sites in the three-dimensional lattice. To connect the percolation behavior to the experimentally applied gate voltage, the average three-dimensional carrier density

$$n_{\mathrm{avg}}(E_F) = \langle n(i,j,k;E_F) \rangle$$

is mapped to gate voltage using a capacitive relation,

$$V_G(E_F) = V_{\mathrm{ref}} + \frac{q\, n_{\mathrm{avg}}(E_F)\, \delta_z}{C_{\mathrm{ox}}}$$

Where C_{ox} is the gate-oxide capacitance per unit area, δ_z is an effective geometrical conversion factor accounting for the finite thickness of the conducting channel, and V_{ref} is a reference voltage offset.

By scanning the E_F, a numerical relation $p(V_G)$ is obtained. The threshold voltage (V_T) is identified as the V_G corresponding to a critical percolation probability (p_c), marking the formation of a continuous three-dimensional conducting network across the channel. This framework demonstrates that the experimentally observed threshold-voltage behavior arises from disorder-induced percolation in a three-dimensional band-edge landscape, rather than from a quantum-confinement-induced shift of the band edge.

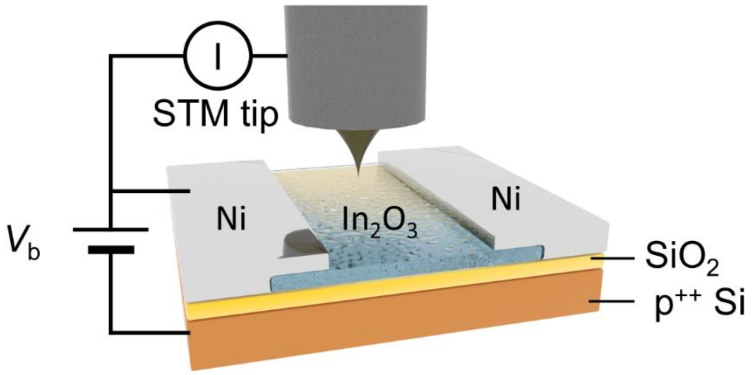

Figure S1. Schematic of the STM measurement setup. A scanning tunneling microscope (STM) tip is positioned above the In_2O_3 channel for local electronic characterization. The In_2O_3 thin film is deposited on a SiO_2/p^{++} Si substrate, with Ni source and drain contacts. A bias voltage (V_b) is applied between the STM tip and the sample while measuring the tunneling current (I). All STM measurements were conducted under an ultra-high vacuum (UHV) environment at a base pressure of 10^{-10} Torr.

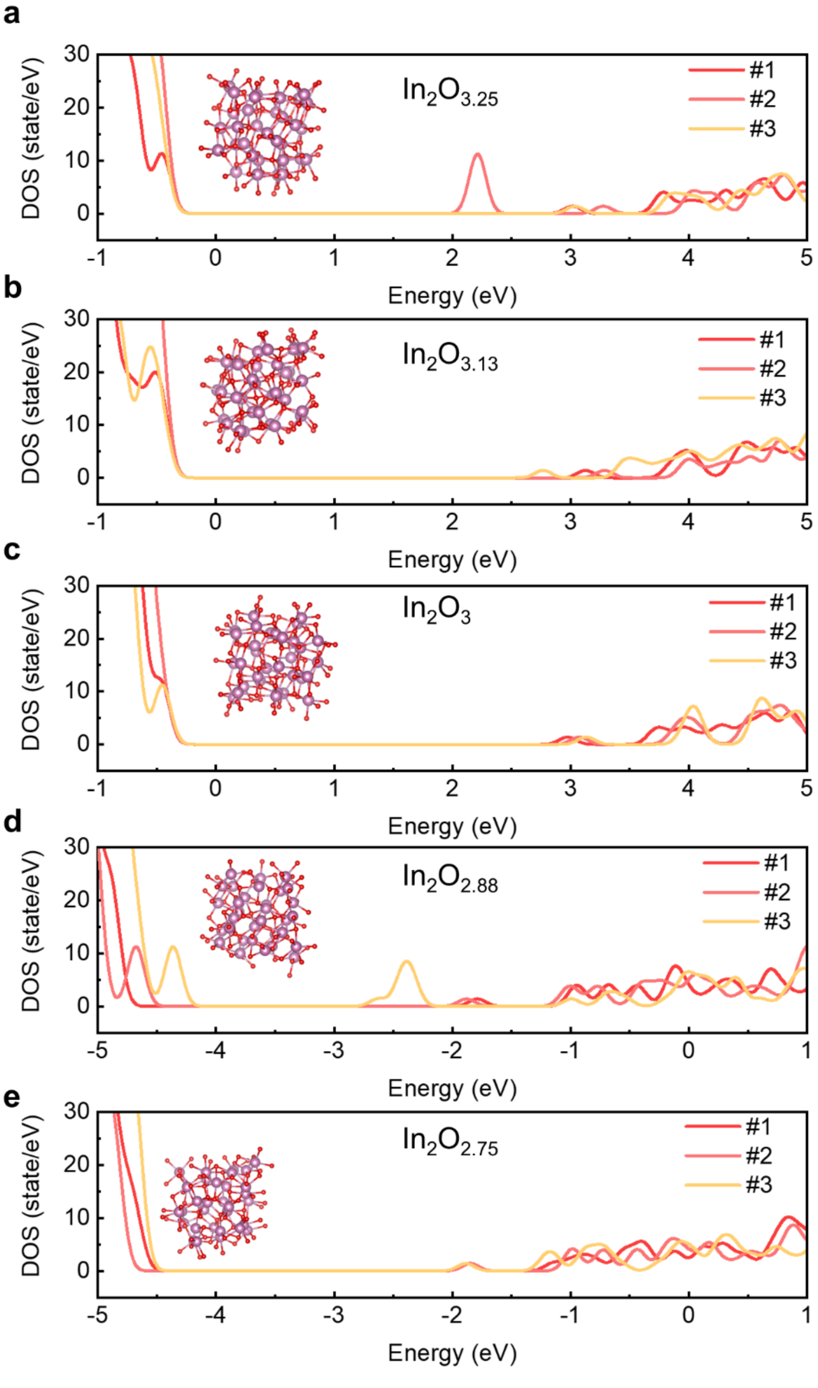

Figure S2. Illustrations of the electronic structures of In_2O_3 under different oxygen contents. (a) $In_{32}O_{52}$, (b) $In_{32}O_{50}$, (c) $In_{32}O_{48}$, (d) $In_{32}O_{46}$, and (e), $In_{32}O_{44}$. Multiple simulations were performed for each sample, maintaining a density of 7.2 g/cm^3. The structures shown were visualized using VESTA/OpenMX software.[12, 13] Due to the intrinsic structural disorder in amorphous systems,

even under identical quenching conditions, each simulation yields slightly different results. To ensure statistical reliability, multiple independent calculations were conducted, leading to the presence of multiple lines in each figure. Label #1, #2 and #3 represent three different independent simulation results under the same conditions.

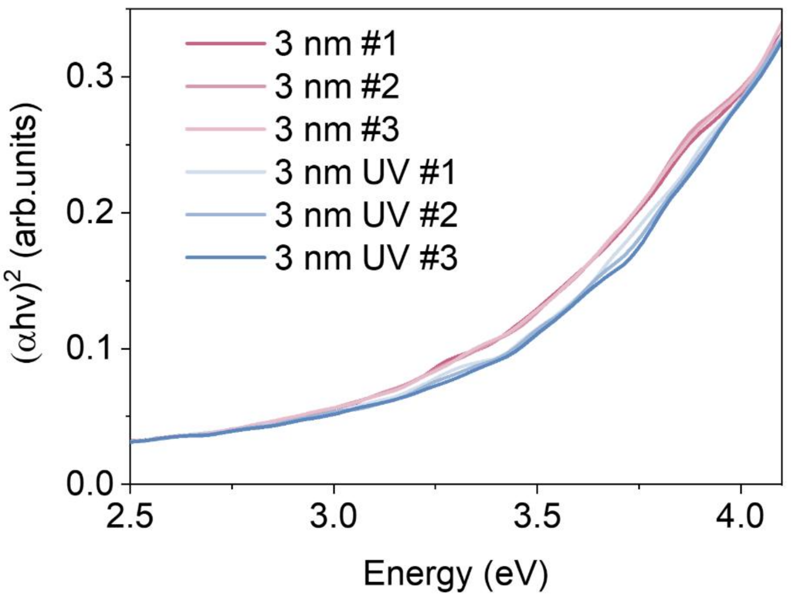

Figure S3. Tauc plot for In_2O_3 films with UV exposure. Absorption spectra plotted as $(\alpha h\nu)^2$ versus photon energy ($h\nu$) for 3 nm In_2O_3 films before and after UV exposure. Labels #1, #2, and #3 correspond to measurements taken at different positions on the same film. Measurements were conducted using a Hitachi U-4100 UV-Visible-NIR Spectrometer with a spot size of approximately 2 cm in diameter.

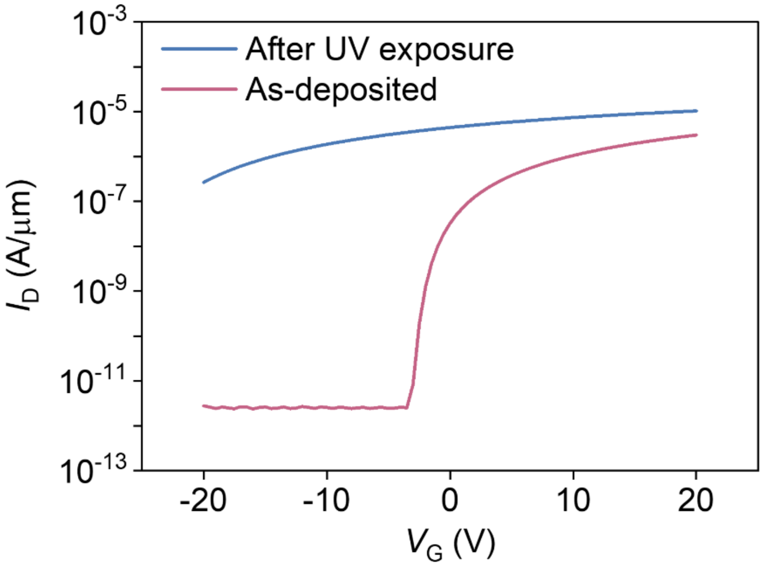

Figure S4. Transfer characteristics of the In_2O_3 device before and after UV exposure. Measurements were performed with a drain voltage (V_D) of 0.1 V, with a channel width of 10 µm and a channel length of 2 µm.

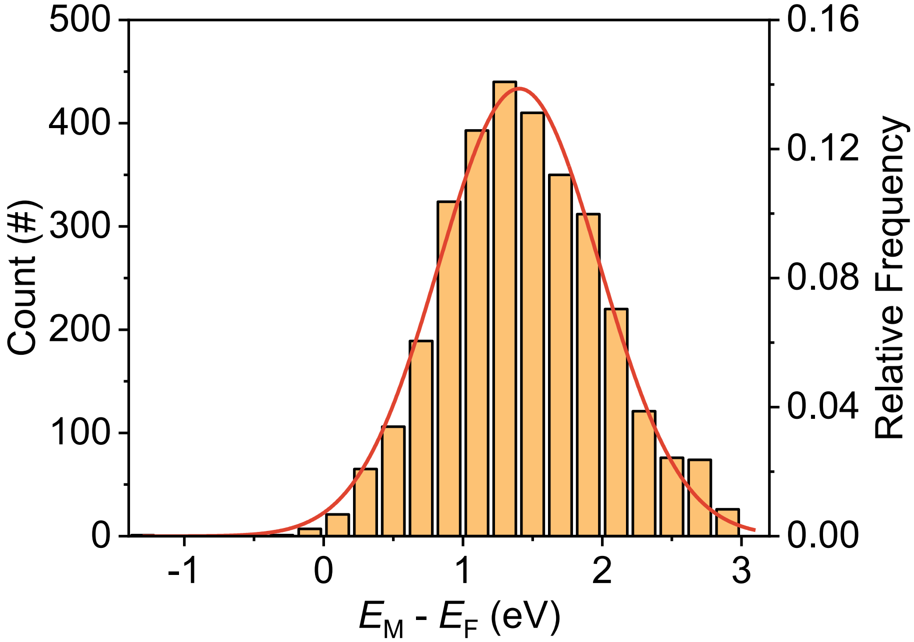

Figure S5. Histogram of the energy barrier (E_M - E_F) distribution. The red line represents a Gaussian fit to the data.

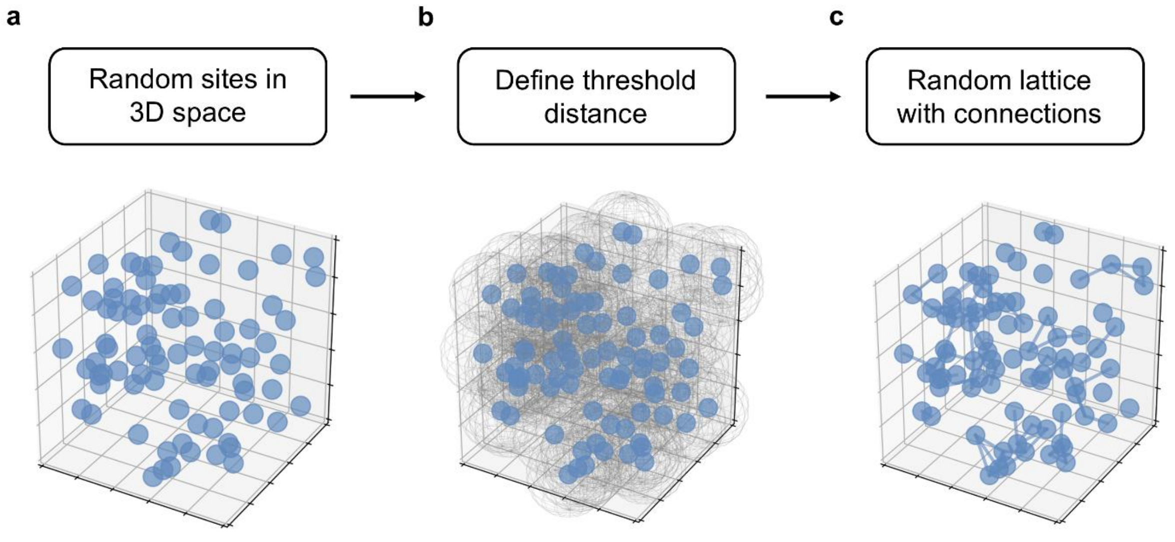

Figure S6. Construction of the random lattice for percolation simulations. (a) Random sites are distributed in a 3D space. (b) A threshold distance (d_{th}) is defined to determine site connectivity (z), forming potential connections between sites. (c) The final random lattice is established, where only sites within the threshold distance are connected, forming a network for percolation analysis.

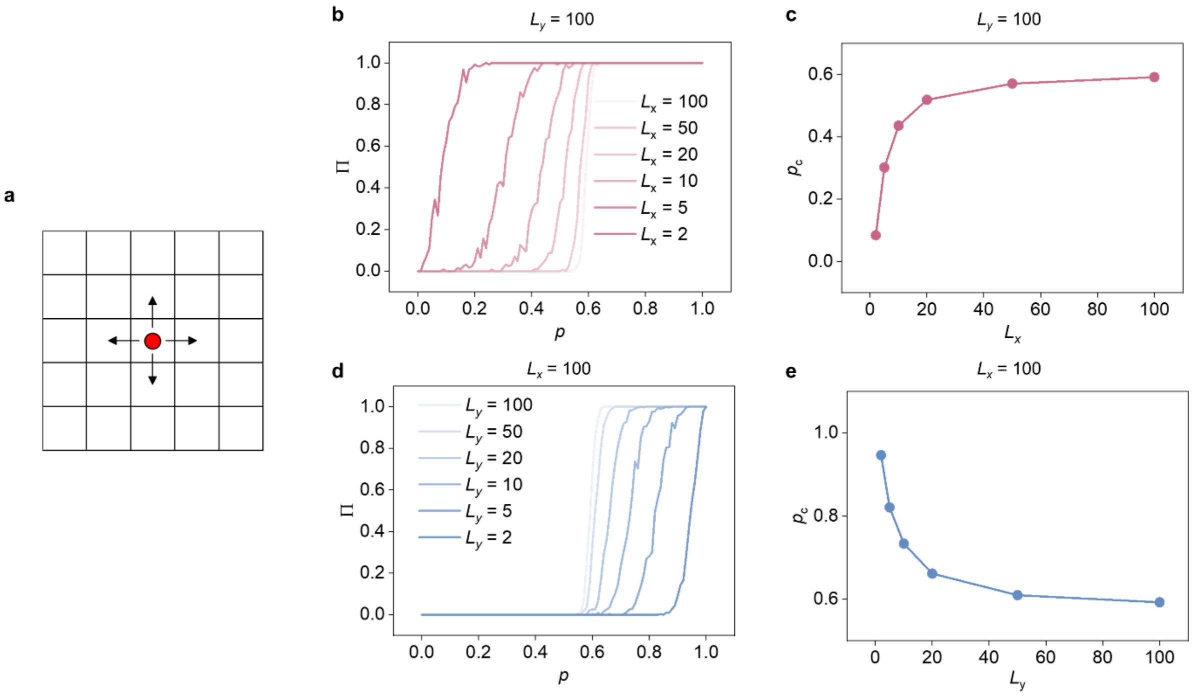

Figure S7. 2D percolation model with nearest-neighbor connectivity. (a) Schematic of a square lattice with each site connected to its four nearest neighbors. (b) Percolation probability (Π) as a function of conducting site fraction (p) for different L_x with L_y = 100. (c) p_c versus L_x with L_y = 100. (d) Π for different L_y with L_x = 100. (e) p_c versus L_y with L_y = 100.

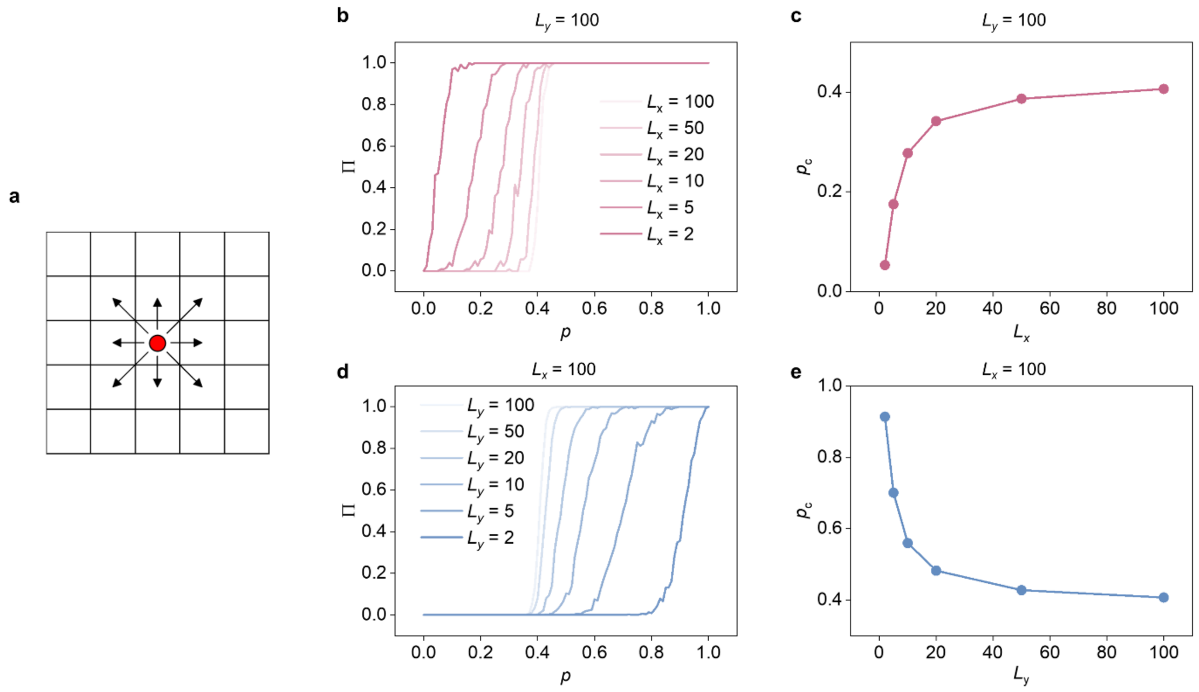

Figure S8. 2D percolation model with next-nearest-neighbor connectivity. (a) Schematic of a square lattice with each site connected to its eight nearest and next-nearest neighbors. (b) Percolation probability (Π) as a function of conducting site fraction (p) for different L_x with L_y = 100. (c) p_c versus L_x with L_y = 100. (d) Π for different L_y with L_x = 100. (e) p_c versus L_y with L_x = 100.

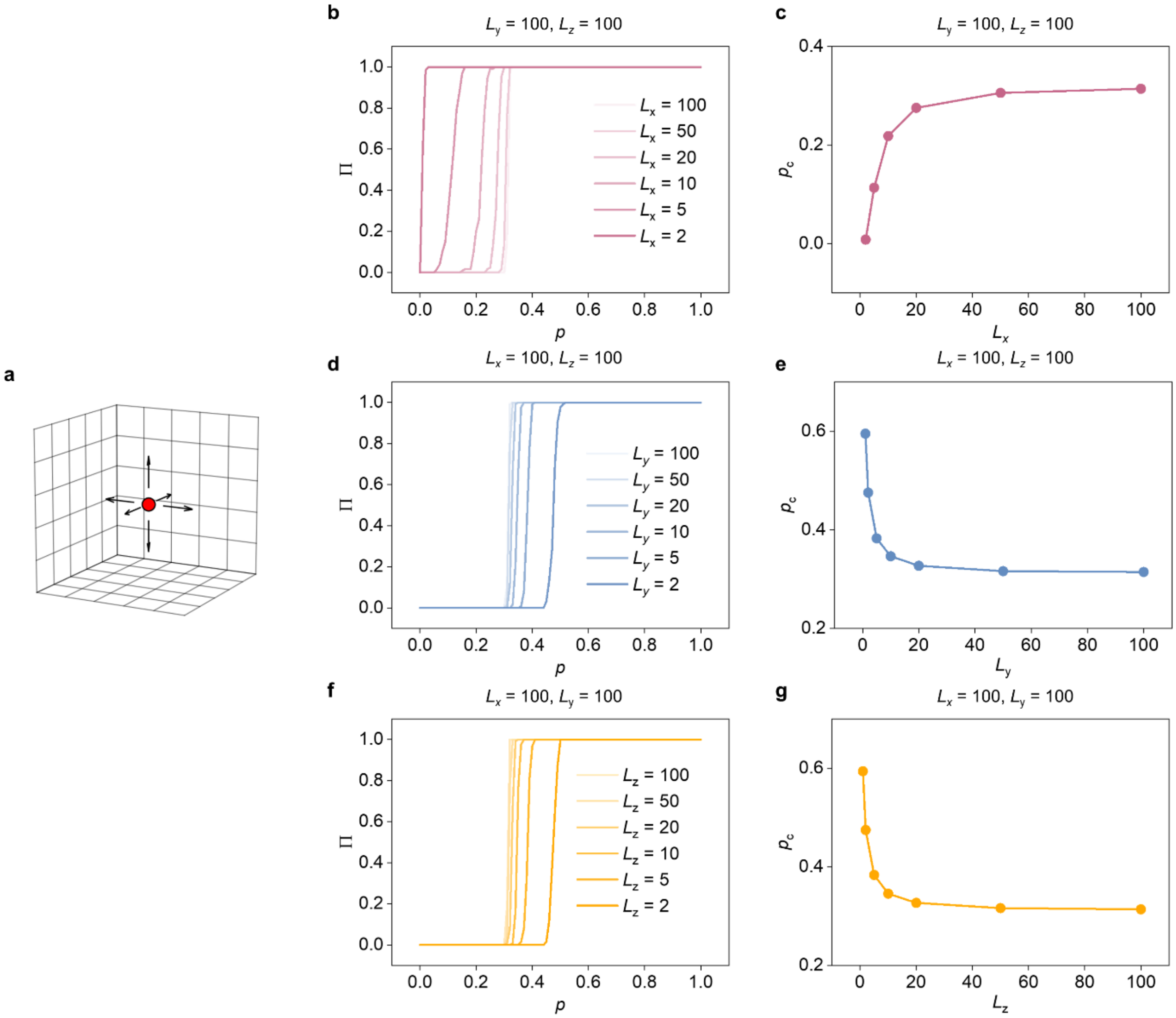

Figure S9. 3D percolation model with nearest-neighbor connectivity. (a) Schematic of a cubic lattice with each site connected to its six nearest neighbors. (b) Percolation probability (Π) as a function of conducting site fraction (p) for different L_x with $L_y = 100$, $L_z = 100$. (c) p_c versus L_x with $L_y = 100$, $L_z = 100$. (d) Π for different L_y with $L_x = 100$, $L_z = 100$. (e) p_c versus L_y with $L_x = 100$, $L_z = 100$. (f) Π for different L_z with L_x, $L_y = 100$. (g) p_c versus L_z with $L_x = 100$, $L_y = 100$.

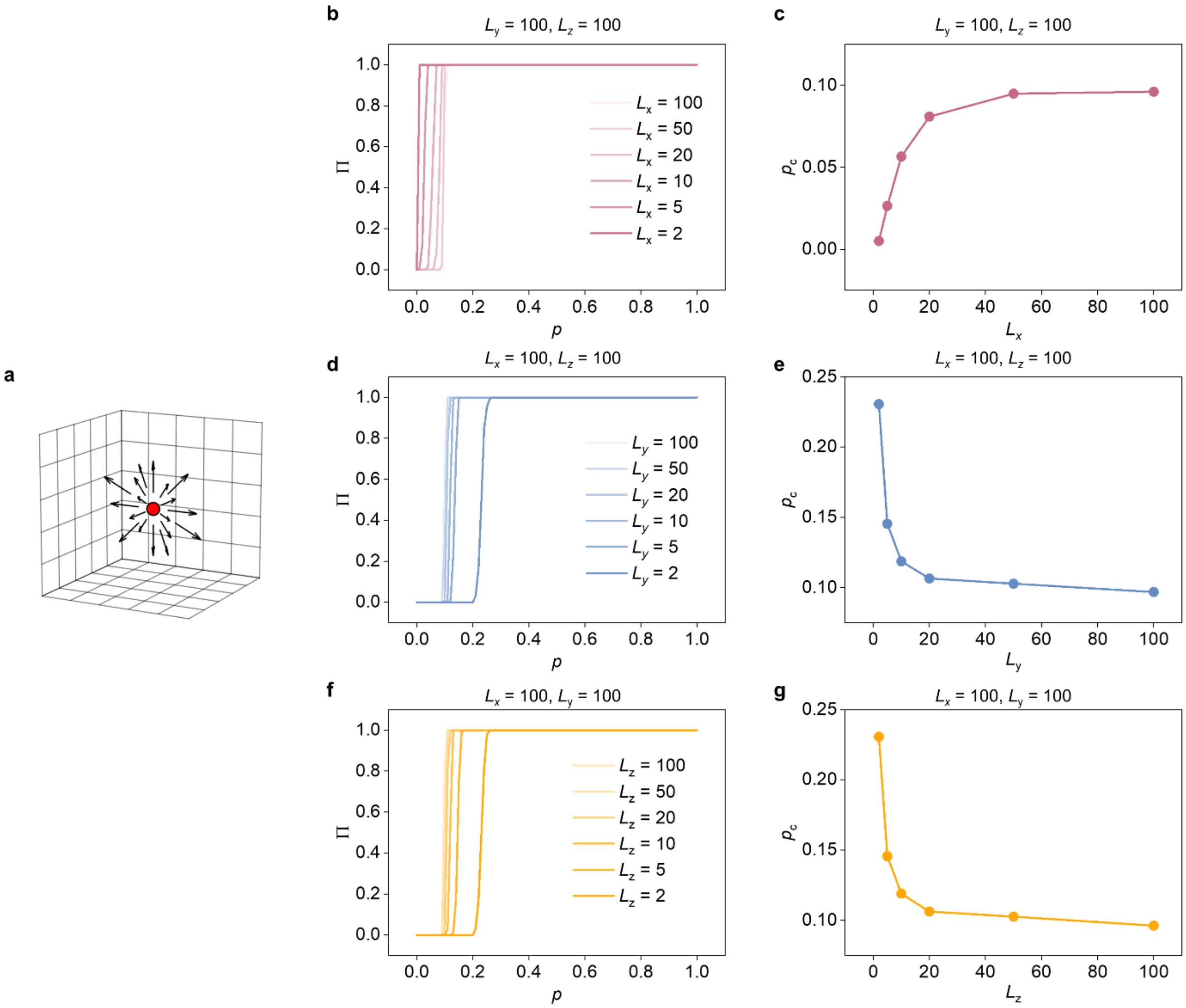

Figure S10. 3D percolation model with next-nearest-neighbor connectivity. (a) Schematic of a cubic lattice with each site connected to its 26 nearest and next-nearest neighbors. (b) Percolation probability (Π) as a function of conducting site fraction (p) for different L_x with $L_y = 100$, $L_z = 100$. (c) p_c versus L_x with $L_y = 100$, $L_z = 100$. (d) Π for different L_y with $L_x = 100$, $L_z = 100$. (e) p_c versus L_y with $L_x = 100$, $L_z = 100$. (f) Π for different L_z with L_x, $L_y = 100$. (g) p_c as versus L_z with $L_x = 100$, $L_y = 100$.

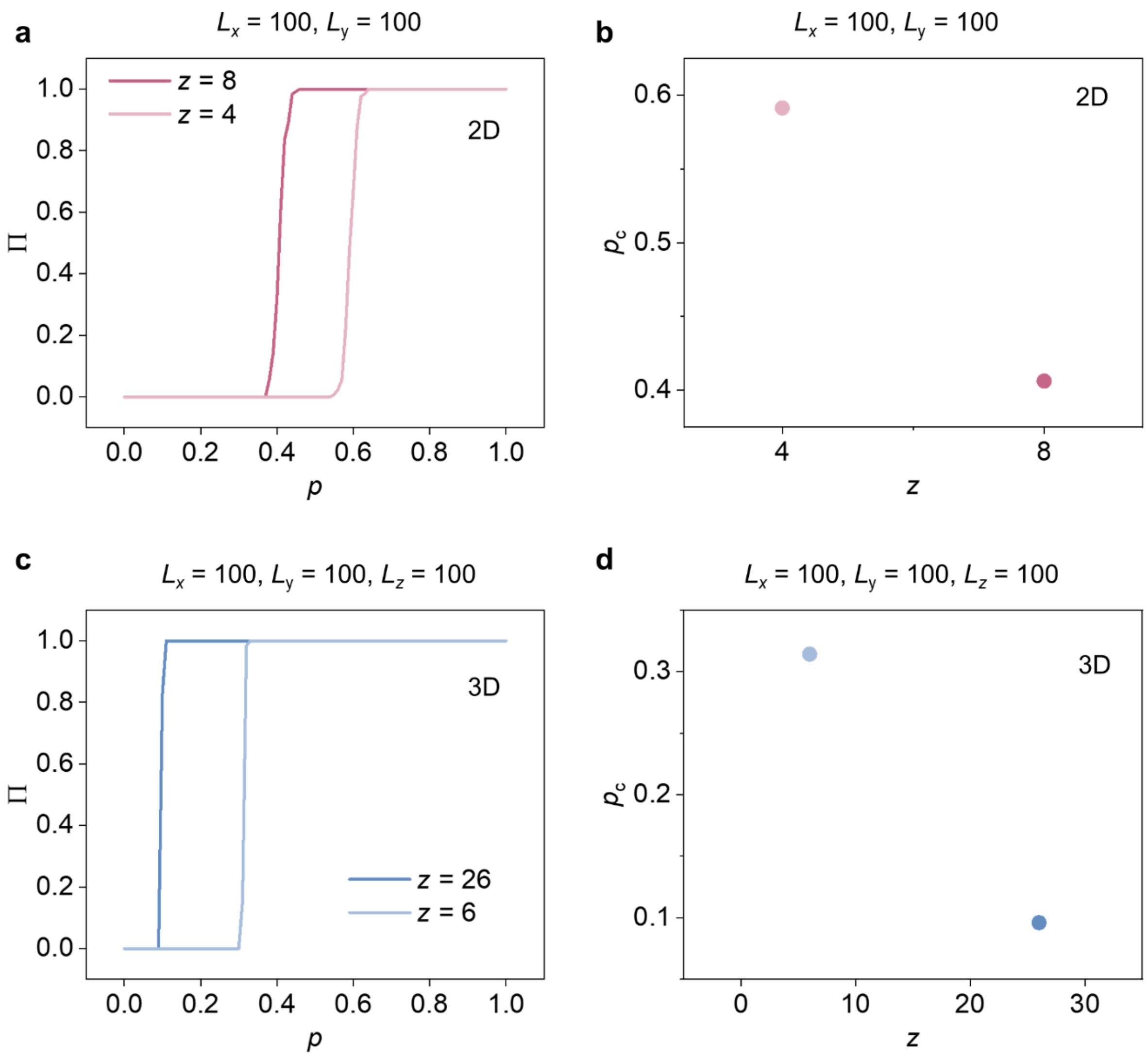

Figure S11. Influence of coordination number (z) on percolation threshold (p_c) in 2D and 3D lattices. (a) Percolation probability (Π) as a function of conducting site fraction (p) for different coordination numbers (z = 4, 8) in 2D lattices. (b) p_c versus z in 2D lattices. (c) Π for different coordination numbers (z = 6, 26) in 3D lattices. (d) p_c versus z in 3D lattices.

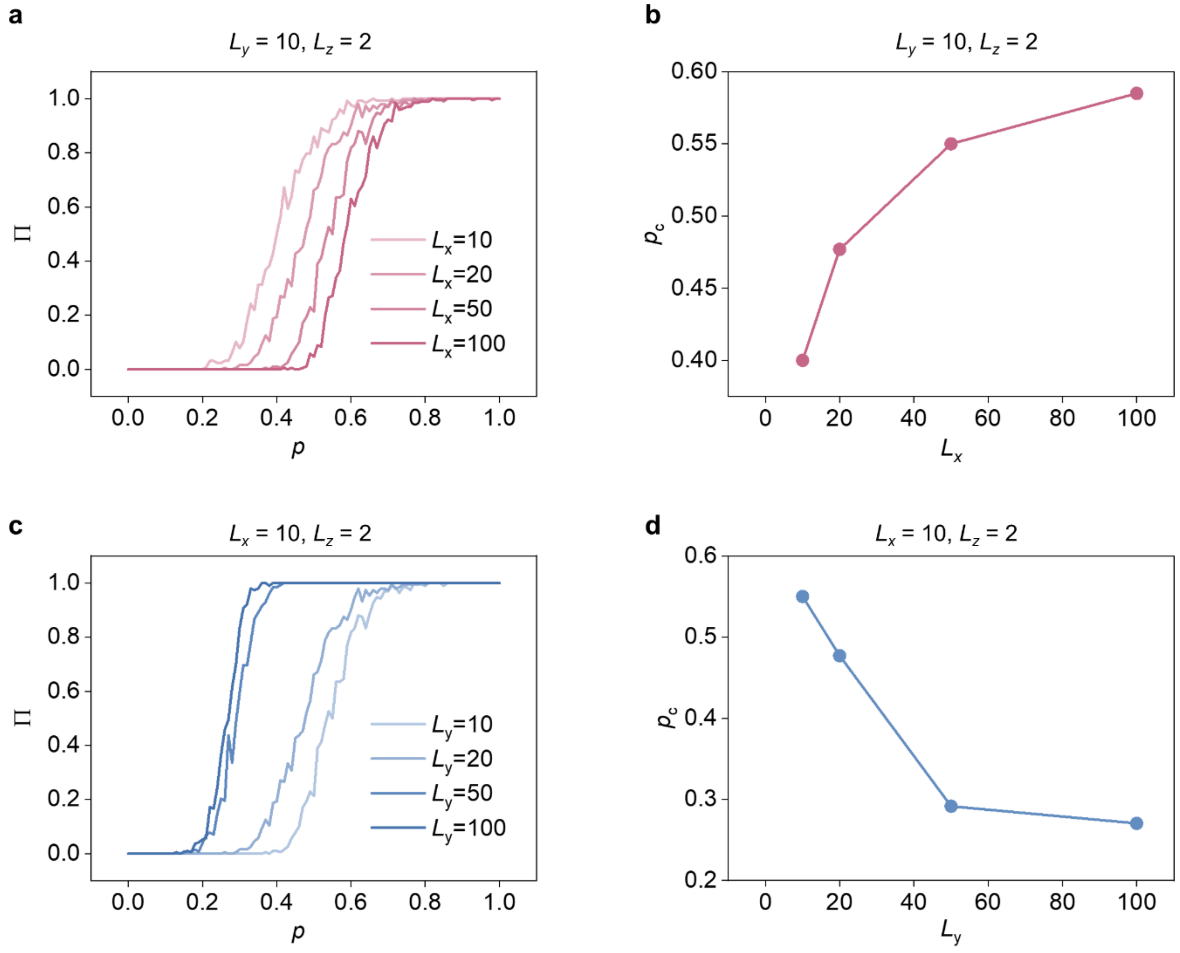

Figure S12. Effect of system size on percolation threshold (p_c) in the constructed random lattice. (a) Percolation probability (Π) as a function of conducting site fraction (p) for different L_x with fixed L_y =10 and L_z = 2. (b) p_c versus L_x with fixed L_y =10 and L_z = 2. (c) Π for different L_y with fixed L_x =10 and L_z = 2. (d) p_c versus L_y with fixed L_x =10 and L_z = 2.

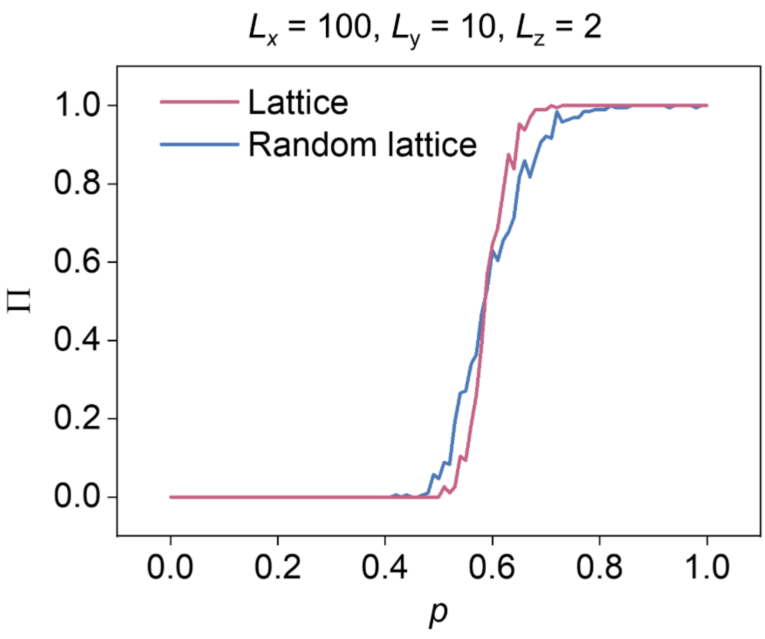

Figure S13. Comparison of percolation behavior between a regular lattice and a random lattice. Percolation probability (Π) as a function of conducting site fraction (p) for a structured lattice and a random lattice with the same dimensions (L_x =100, L_y =10 and L_z = 2)

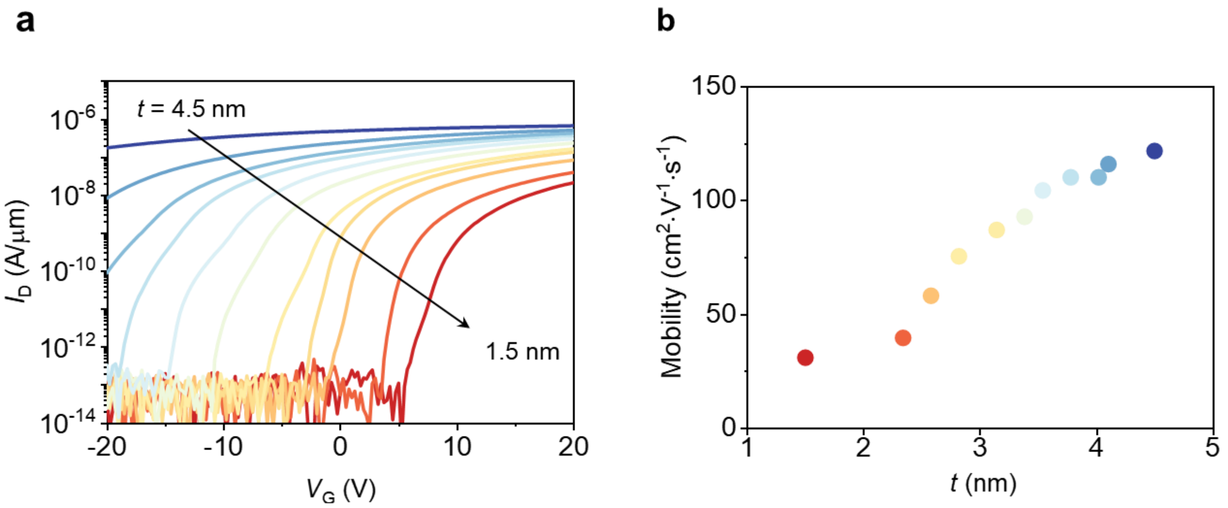

Figure S14. Transfer characteristics and mobility degradation with decreasing film thickness in n-type In_2O_3 transistors. (a) Transfer characteristics of In_2O_3 transistors, measured on devices with $l = w = 50$ µm and at $V_D = 0.1$ V, as channel thickness is reduced from 4.5 nm (blue) to 1.5 nm (red). (b) Corresponding field-effect mobility extracted from the curves in (a).

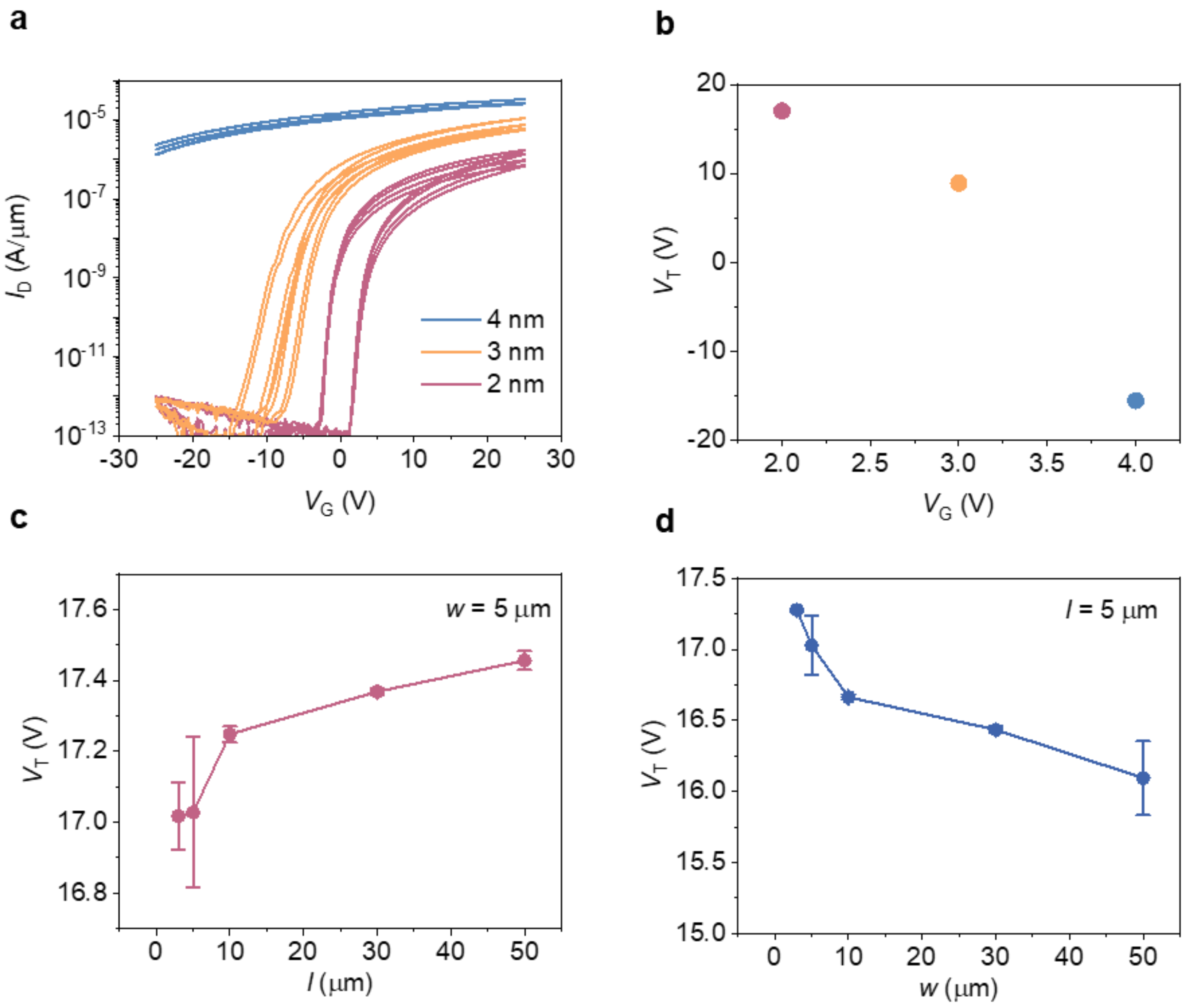

Figure S15. Dimensional scaling effect in sputtered amorphous In_2O_3 transistors. (a) Transfer characteristics for multiple devices with varying channel thicknesses (4, 3, and 2 nm). (b) Corresponding V_T as a function of channel thickness. (c) V_T as a function of l for 2 nm thick devices (w = 5 μm). (d) V_T as a function of w for 2 nm thick devices (l = 5 μm).

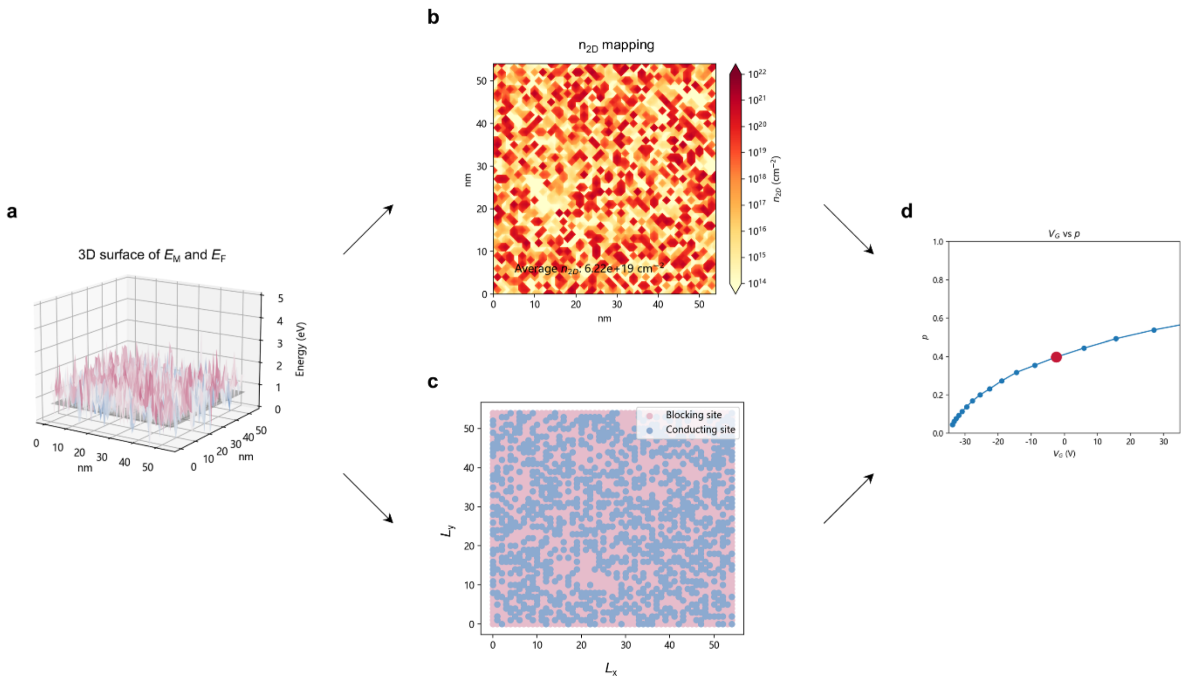

Figure S16. The correlation between the percolation threshold (p_c) and the threshold voltage (V_T) for a quasi-2D semiconductor system. (a) 3D surface plot of the Gaussian distributed mobility edge (E_M) with Femi level (E_F) = 0.7 eV. (b) Carrier concentration distribution (n_{2D}) map with E_F = 0.7 eV. (c) Conducting vs blocking site map with E_F = 0.7 eV, where blue represents conducting sites and pink represents blocking sites. (d) Plot of the gate voltage (V_G) vs conducting fraction (p). The red point represents the correlation of V_G and p with E_F = 0.7 eV.

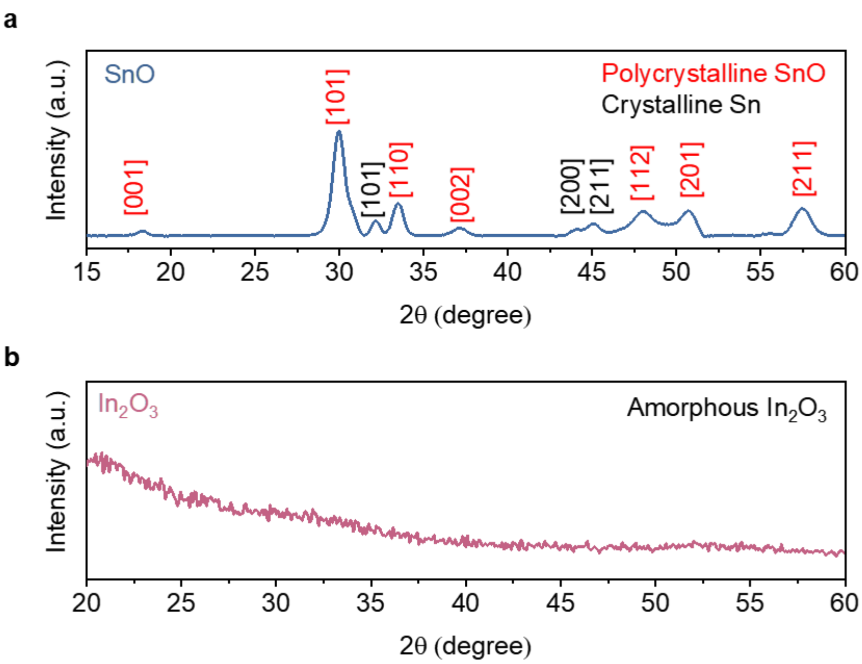

Figure S17. X-ray diffraction (XRD) patterns of (a) polycrystalline SnO and (b) amorphous In_2O_3.

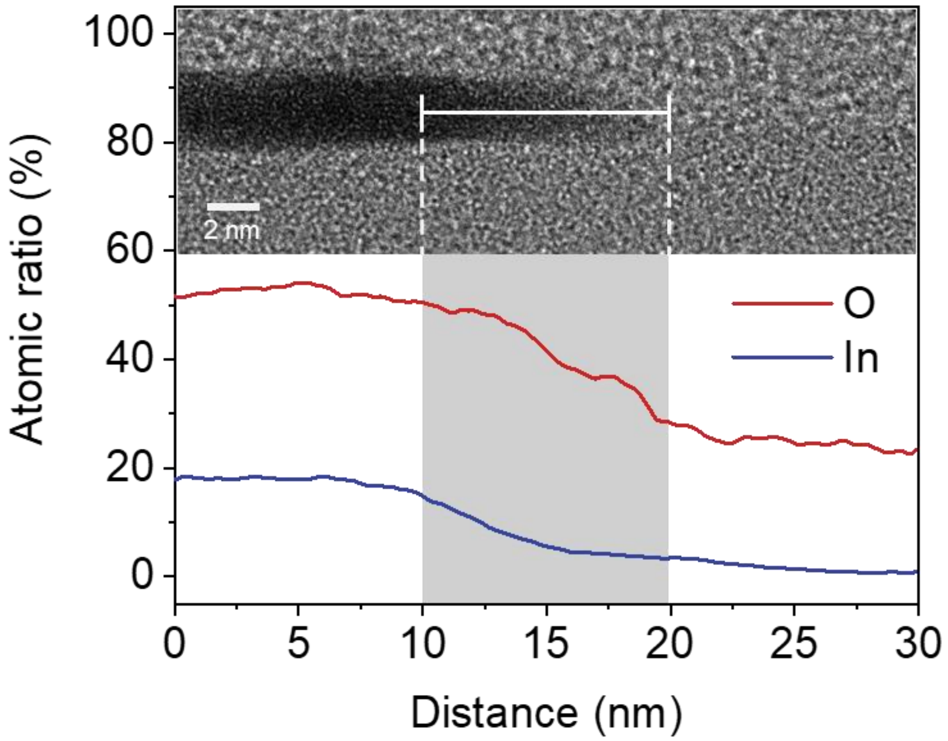

Figure S18. Microstructural and chemical analysis of the etched In_2O_3 channel edge. EDS line-scan profiles of In and O extracted from the highlighted region in the inset TEM image.

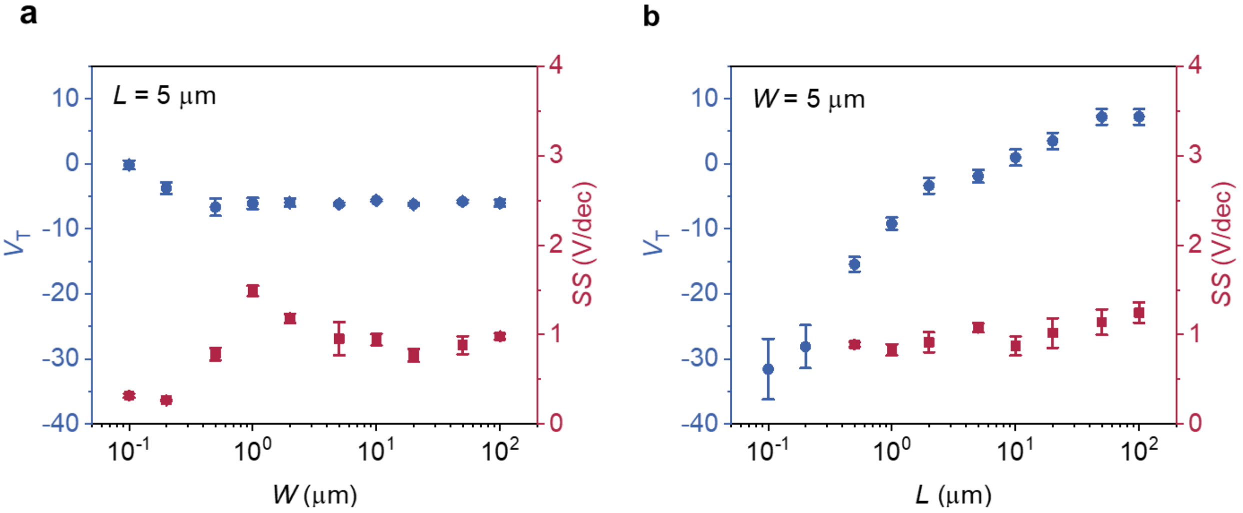

Figure S19. Correlation between threshold voltage (V_T) and subthreshold swing (*SS*) with varying lateral dimensions. (a) V_T (blue, left axis) and SS (red, right axis) as a function of channel length (*l*) for In_2O_3 transistors with a fixed width of 5 µm. (b) V_T and *SS* as a function of channel width (*w*) for devices with a fixed length of 5 µm. All devices share a fixed thickness of 2.5 nm, chosen to ensure optimal conditions for reliable *SS* extraction. The error bars represent the standard deviation from measurements on multiple devices. *SS* data for V_T < -20 V are omitted due to unreliable extraction.

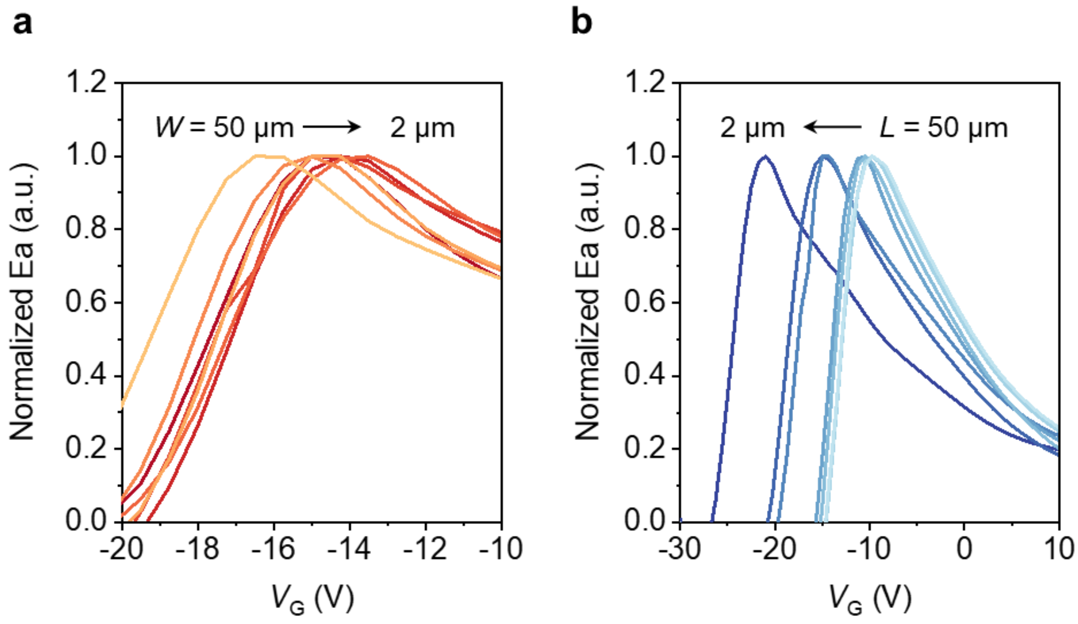

Figure S20. Geometric modulation of activation energy**.** V_G dependence of E_a for In_2O_3 transistors with (a) varying *w* at a fixed *l* = 5 μm and (b) varying channel length *l* at a fixed *w* = 5 μm.

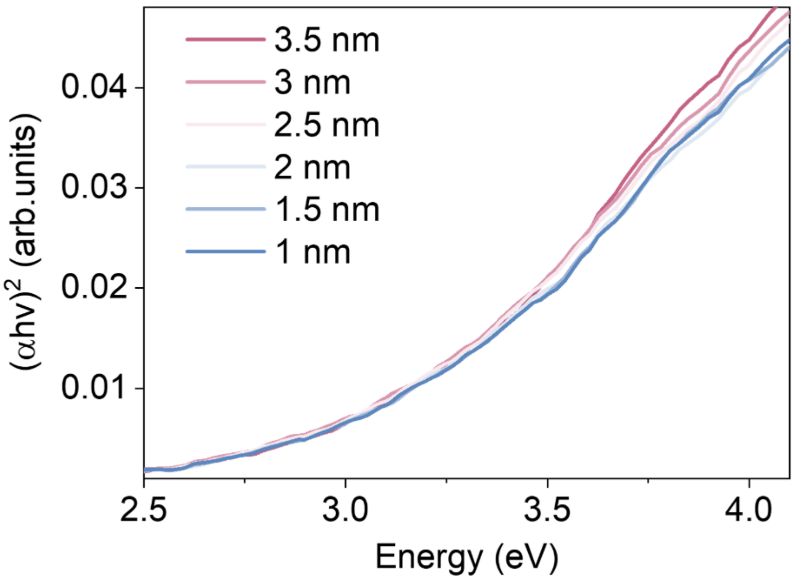

Figure S21. Tauc plot for In_2O_3 films of varying thicknesses. Absorption spectra plotted as $(\alpha h\nu)^2$ versus photon energy ($h\nu$) for films with thicknesses ranging from 1 to 3.5 nm. The optical bandgap was estimated from the linear region of each curve. Measurements were conducted using a Hitachi U-4100 UV-Visible-NIR Spectrometer with a spot size of approximately 2 cm in diameter.

References

(1) Marx, D.; Hutter, J. Ab initio molecular dynamics: Theory and implementation. *Modern methods and algorithms of quantum chemistry* **2000**, *1* (301-449), 141.

(2) Kresse, G.; Joubert, D. From ultrasoft pseudopotentials to the projector augmented-wave method. *Phys. Rev. B.* **1999**, *59* (3), 1758.

(3) Kresse, G.; Furthmüller, J. Efficient iterative schemes for ab initio total-energy calculations using a plane-wave basis set. *Phys. Rev. B.* **1996**, *54* (16), 11169.

(4) Perdew, J. P.; Burke, K.; Ernzerhof, M. Generalized gradient approximation made simple. *Phys. Rev. Lett.* **1996**, *77* (18), 3865.

(5) Blöchl, P. E. Projector augmented-wave method. *Phys. Rev. B.* **1994**, *50* (24), 17953.

(6) Heyd, J.; Peralta, J. E.; Scuseria, G. E.; Martin, R. L. Energy band gaps and lattice parameters evaluated with the Heyd-Scuseria-Ernzerhof screened hybrid functional. *The Journal of chemical physics* **2005**, *123* (17).

(7) Heyd, J.; Scuseria, G. E.; Ernzerhof, M. Hybrid functionals based on a screened Coulomb potential. *The Journal of chemical physics* **2003**, *118* (18), 8207-8215.

(8) Medvedeva, J. E.; Zhuravlev, I.; Burris, C.; Buchholz, D.; Grayson, M.; Chang, R. Origin of high carrier concentration in amorphous wide-bandgap oxides: Role of disorder in defect formation and electron localization in In_2O_{3-x}. *J. Appl. Phys.* **2020**, *127* (17).

(9) Rosén, J.; Warschkow, O. Electronic structure of amorphous indium oxide transparent conductors. *Phys. Rev. B Condens. Matter* **2009**, *80* (11), 115215.

(10) Urata, S.; Nakamura, N.; Kim, J.; Hosono, H. Role of hydrogen-doping for compensating oxygen-defect in non-stoichiometric amorphous In_2O_{3-x}: Modeling with a machine-learning potential. *J. Appl. Phys.* **2023**, *134* (11).

(11) Fishchuk, I. I.; Kadashchuk, A.; Bhoolokam, A.; de Jamblinne de Meux, A.; Pourtois, G.; Gavrilyuk, M.; Köhler, A.; Bässler, H.; Heremans, P.; Genoe, J. Interplay between hopping and band transport in high-mobility disordered semiconductors at large carrier concentrations: The case of the amorphous oxide InGaZnO. *Phys. Rev. B.* **2016**, *93* (19), 195204.

(12) Lee, Y.-T.; Ozaki, T. OpenMX Viewer: A web-based crystalline and molecular graphical user interface program. *Journal of Molecular Graphics and Modelling* **2019**, *89*, 192-198.

(13) Momma, K.; Izumi, F. An integrated three-dimensional visualization system VESTA using wxWidgets. In *atelier de diffraction sur poudre Seventh Canadian Powder Diffraction Workshop*, 2006; p 106.
